# The MLL1 trimeric catalytic complex is a dynamic conformational ensemble stabilized by multiple weak interactions

**DOI:** 10.1093/nar/gkz697

**Published:** 2019-08-10

**Authors:** Lilia Kaustov, Alexander Lemak, Hong Wu, Marco Faini, Lixin Fan, Xianyang Fang, Hong Zeng, Shili Duan, Abdellah Allali-Hassani, Fengling Li, Yong Wei, Masoud Vedadi, Ruedi Aebersold, Yunxing Wang, Scott Houliston, Cheryl H Arrowsmith

**Affiliations:** 1 Princess Margaret Cancer Centre and Department of Medical Biophysics, University of Toronto, ON M5G 2M9, Canada; 2 Department of Anesthesia, Sunnybrook Health Sciences Centre, Toronto, ON M4N 3M5, Canada; 3 Structural Genomics Consortium, University of Toronto, 101 College Street, MaRS Centre, South Tower, Toronto, ON M5G 1L7, Canada; 4 Department of Biology, Institute of Molecular Systems Biology, ETH Zürich, 8093 Zürich, Switzerland; 5 The Small-Angel X-ray Scattering Core Facility, Center for Cancer Research of National Cancer Institute, Frederick National Laboratory for Cancer Research, Leidos Biomedical Research, Inc. Frederick, MD 21702, USA; 6 Department of Pharmacology and Toxicology, University of Toronto, Toronto, ON, M5S 1A8, Canada; 7 Faculty of Science, University of Zürich, 8057 Zürich, Switzerland

## Abstract

Histone H3K4 methylation is an epigenetic mark associated with actively transcribed genes. This modification is catalyzed by the mixed lineage leukaemia (MLL) family of histone methyltransferases including MLL1, MLL2, MLL3, MLL4, SET1A and SET1B. The catalytic activity of this family is dependent on interactions with additional conserved proteins, but the structural basis for subunit assembly and the mechanism of regulation is not well understood. We used a hybrid methods approach to study the assembly and biochemical function of the minimally active MLL1 complex (MLL1, WDR5 and RbBP5). A combination of small angle X-ray scattering, cross-linking mass spectrometry, nuclear magnetic resonance spectroscopy and computational modeling were used to generate a dynamic ensemble model in which subunits are assembled via multiple weak interaction sites. We identified a new interaction site between the MLL1 SET domain and the WD40 β-propeller domain of RbBP5, and demonstrate the susceptibility of the catalytic function of the complex to disruption of individual interaction sites.

## INTRODUCTION

Post-translational modifications on histone tails are key epigenetic signals for regulation of chromatin structure and gene expression. H3K4 methylation is a complex, dynamic process that is strongly correlated with actively transcribed genes or those that are in a poised or bivalent state (1). Mono-, di- and trimethlyated species of H3K4 exhibit a gradient distribution with respect to transcription start sites (TSSs); H3K4me3 is most abundant close to TSSs and in promoter regions, whereas H3K4me2/me1 marked histones are enriched further up- and downstream (2). H3K4 methylation is catalyzed by the MLL/SET1 family of histone methyltransferases (3,4), through their evolutionarily conserved SET domain (5,6). The founding member of this family of H3K4 methyltransferases is the yeast SET1 protein (7,8). In mammals, methylation of H3K4 is carried out by a family of six proteins: MLL (mixed lineage leukemia protein)1 to MLL4, SET1A and SET1B (9–15). The MLL proteins play crucial roles in embryonic development and hematopoiesis through transcriptional regulation of the clustered homeobox (*Hox*) genes and other genes important for developmental regulation (10,16–19). Deletion of MLL1 and MLL2 can lead to severe defects in embryonic development in mice (18,20). The *MLL1* gene is frequently rearranged in human acute leukemia in both adults and children (21–23). Recently, studies have identified inactivating mutations in MLL3 and MLL4 in different types of human tumors (24–27), as well as in Kabuki syndrome (28).

The catalytic activity of MLL/SET family members are dependent to varying degrees on the presence of additional evolutionarily conserved protein subunits, RbBP5, WDR5, ASH2L and DYP30, which together form the core complexes of MLL enzymes (29–34). A minimal core enzyme can be reconstituted with the C-terminal SET domain fragment of MLLs and at least two of the other subunits (29,31,35). In studies of these reconstituted core enzymes, MLL1 appears to be unique among the family members in its requirements for, and interactions with other subunits. For example, in relation to other MLLs, the catalytic activity of MLL1 is most strongly stimulated by WDR5 (31,36,37), whereas it binds with the least affinity and is only weakly stimulated by the RbBP5–ASH2L heterodimer (35).

Crystallographic studies of MLL3 support a model in which the RbBP5–ASH2L heterodimer stabilizes the catalytically active conformations of MLL2/3/4 through interactions with conserved surfaces on their SET domain (35). However, it was suggested that two key variant residues on this surface of MLL1 dramatically weakened the interaction between MLL1 and RbBP5–ASH2L relative to that of other MLL members, thereby increasing the dependence of MLL1 on WDR5 (35). The unique dependence of MLL1 activity on WDR5 may be of therapeutic relevance, as we and others have shown that pharmacological targeting of the MLL interaction site on WDR5 can functionally antagonize MLL1 in cancers that are dependent on MLL1 activity (38–40).

While there are several structures of WDR5 bound to MLL and RbBP5 peptides (37,41–44), as well as a crystal structure of the apo-SET domain (45) of MLL1 and a 24 Å resolution cryo-EM model of the homologous yeast COMPASS (46), an atomic level picture of a functional MLL1 catalytic complex is still lacking. There is evidence of a hierarchical organization, wherein WDR5 and RbBP5 jointly interact with MLL1 to form a stable species (29,31,34), which we refer to as the ‘minimal catalytic complex’. This trimer can serve as a scaffold for the association of ASH2L and DPY30 (29).

Here, we report a hybrid methods study of MLL1 and its catalytic core components in solution. Using small angle X-ray scattering (SAXS), cross-linking mass spectrometry (XL-MS), nuclear magnetic resonance (NMR) spectroscopy and computational modeling we derived a dynamic ensemble model for the WDR5–RbBP5–MLL1 complex, and identify a new interaction site between the MLL1 SET domain, and the N-terminal WD40 repeat domain of RbBP5. Our data support the notion that the functional MLL1 enzyme comprises a collection of weak but specific interactions, and that the disruption of individual interactions can have significant destabilizing effects on the entire complex.

## MATERIALS AND METHODS

### Cloning of MLL1, WDR5 and RbBP5 constructs

The coding regions for human MLL1_RBS-SET_ (residues 3785–3969) and MLL1_WIN-SET_(residues 3745–3969) were polymerase chain reaction (PCR)-amplified and subcloned into the pET28GST-LIC vector (GeneBank ID: EF456739). We generated two mutants of the MLL_WIN-SET_ construct from the wild-type clone using QuikChange PCR mutagenesis kit (Agilent): (i) MLL1_RBS-SET_7D (where residues 3786–3792 were deleted) and (ii) MLL1_RBS-SET_3M (which has Q3787V, P3788L and Y3791G mutations). RbBP5 constructs of different lengths (comprising residues 10–340, 10–410, 320–410, 340–538 and 1–538) and WDR5_WD40_ (residues 24–334) were subcloned into the pET28-MHL vector (GeneBank ID: EF456738). For the characterization of the dimeric complexes, and reconstitution of the MLL1 trimeric complex, the following construct pairs were cloned into the pFastBac Dual expression vector (Thermo Fisher Scientific): (i) full-length WDR5 and His-tagged MLL1_WIN-SET_, and (ii) full-length WDR5 and His-tagged RbBP5.

### Protein preparation

Individual components of the MLL1 complex were expressed in *Escherichia coli* and purified using an N-terminal GST-tag (for MLL1) or His-tag (for WDR5 and RbBP5). We found MLL1_RBS-SET_ to be better behaved and more stable than MLL1_WIN-SET_. Therefore, we used the MLL1_RBS-SET_ construct whenever possible. The characterization of complexes involving both MLL1 and WDR5 required the use of MLL1_WIN-SET_. The dimeric and trimeric complexes of MLL1 used for SAXS and cross-linking studies were expressed in Sf9 cells. The dimeric complex of WDR5–MLL1_WIN-SET_ and WDR5–RbBP5 were purified using TALON affinity resin (Clontech), followed by gel filtration chromatography. Purified dimeric complexes were incubated together on ice for 2 h to reconstitute the trimeric complex, which was subsequently purified and recovered by gel filtration chromatography. Detailed procedures are described in the ‘Supplementary Data’ section.

### SAXS data collection, analysis and modeling

SAXS measurements were carried out at the beamline 12-ID-C of the Advanced Photon Source, Argonne National Laboratory. The energy of the X-ray beam was 18 Kev (wavelength *λ* = 0.6888 Å), and two setups (small- and wide- angle X-ray scattering, SAXS and WAXS) were used in which the sample to charge-coupled device detector (MAR research, Hamburg) distance were adjusted to achieve scattering *q* values of 0.006 < *q* < 2.3Å^−1^, where *q* = (4π/λ)sinθ and 2θ is the scattering angle. Data were analyzed using the program PRIMUS (ATSAS package, EMBL (47)). Detailed descriptions of SAXS data collection, analysis and modeling protocols, are provided in the Supplementary Data.

### Chemical cross-linking mass spectrometry

The reconstituted trimer of WDR5, RbBP5 and MLL1_WIN-SET_ was cross-linked at a concentration between 12 μM and 16 μM, with 1 mM of isotopically coded disuccinimidyl suberate (DSS-d_0_,DSS-d_12_) as described previously (48). Protease digestion was carried out with LysC and trypsin. After acidification, cross-linked peptides were purified on C18 cartridges and enriched by size-exclusion chromatography (SEC). SEC fractions were analyzed in duplicate on an LC-MS (Easy-nLC 300; Orbitrap LTQ XL). For complete details, refer to Supplementary Data.

### NMR spectroscopy

NMR spectra were collected at 25°C on a Bruker spectrometer operating at 800 MHz, and equipped with a cryoprobe. Samples contained 5% D_2_O with protein concentrations ranging from 100 to 350 μM. For the assignment of backbone resonances of WDR5_WD40_, a triply-labeled (^15^N/^13^C/^2^H) sample was prepared and conventional triple-resonance backbone spectra were acquired as described previously (49), using the ABACUS approach (50). (^1^H-^15^N)-TROSY titrations of ^15^N-labeled WDR5_WD40_ were performed by adding aliquots of peptides corresponding to the MLL1 WIN motif (GSARAEVHLRKS—i.e. MLL1_3762-3773_) and RbBP5 WBM motif (EDEEVDVTSV—i.e. RbBP5_371-380_) at molar ratios ranging from 1:1 to 1:7. Weighted chemical shift displacements were calculated using the formula: Δppm = [(δ_NH_)^2^+(δ_N_/5)^2^]^1/2^. Spectra were processed with NMR Pipe (51) and analyzed with SPARKY (52).

### GST Pull-down experiments

Recombinant purified MLL1-GST proteins were incubated with various RbBP5 constructs (in an assay buffer containing 20 mM TRIS pH 7.7, 150 mM NaCl, 10 μM ZnCl_2_, 5 mM β-mercaptoethanol, 5 mM dithiothreitol (DTT), and 1 mM phenylmethanesulfonyl fluoride (PMSF)) in a 1:2 molar ratio at 4°C for 1 h. Proteins were then incubated with 100 μL of glutathione-Sepharose beads (GE Healthcare) for an additional 1 h. The mixture was transferred to a micro-column and was extensively washed with assay buffer. Bound proteins were eluted with 30 mM reduced glutathione, and detected by sodium dodecyl sulphate-polyacrylamide gelelectrophoresis (SDS-PAGE) and Coomassie staining.

### Biolayer Interferometry

The interaction between various RbBP5 constructs with GST-tagged MLL_RBS-SET_ and WDR5 was measured using the Octet Red System (Forte Bio). All experiments were performed using phosphate-buffered saline containing 0.2 mg/ml bovine serum albumin and 0.1% (v/v) Tween-20, in a 96-well plate with 200 μL in each well and constant shaking (1000 rpm). GST-tagged constructs were loaded onto anti-GST antibody-coated biosensors (Forte Bio), and the sensors were washed for an extended period in the buffer. Loaded sensors were then incubated with RbBP5 constructs at different concentrations before discharge into separate buffer wells. The binding affinity was determined by steady-state analysis using the program Gnuplot.

### Histone methyltransferase assay

Activity assays were performed in 50 mM Tris–HCl, pH 8.0, 5 mM DTT and 0.01% Triton X-100, using 5 μM ^3^H-SAM and 5 μM Biotin-H3_1-25_. Increasing concentrations of RbBP5 were added to 200 nM of WDR5–MLL1_WIN-SET_ (with either wild-type or mutant MLL1). All reactions were incubated for 90 min at room temperature and a scintillation proximity assay (SPA) was used to determine activities. Experiments were performed in triplicate. For assays with OICR-9429, increasing concentrations of the compound was incubated with 200 nM WDR5–MLL1_WIN-SET_ for 20 min before adding 400 nM RbBP5.

## RESULTS AND DISCUSSION

### SAXS data reveal solution ensembles for WDR5, RbBP5 and MLL1_RBS-SET_

**Figure 1.**
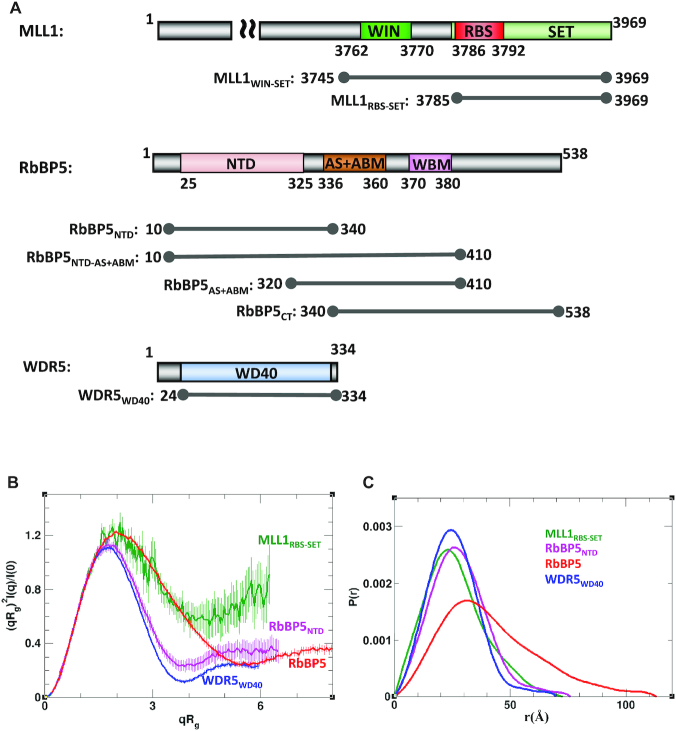
Individual components of the WDR5–RbBP5–MLL1 complex. (**A**) Schematic representation of MLL1, RbBP5 and WDR5 domain organization and constructs used in this study. For clarity, only the C-terminal region is displayed for MLL1. WIN: WDR5 interacting motif as previously defined (41); RBS: RbBP5 binding site as defined in this study; SET: catalytic methyltransferase domain; NTD: N-terminal domain; CT: C-terminus; AS+ABM: activation segment and ASH2L binding motif as defined in (35); WBM: WDR5 binding motif (43). (**B**) SAXS-derived Rg-based Kratky plots indicate that WDR5_WD40_ (blue) and RbBP5_NTD_ (magenta) are globular, while RbBP5 (red) and MLL1_RBS-SET_ (green) exhibit some degree of flexibility. The error bars show propagated experimental errors. (**C**) Normalized pair distance distribution functions P(r) calculated from experimental SAXS data with GNOM.

To model catalytically active MLL1 complexes, we first collected reference solution data for the individual subunits including the SET domain of MLL1, the WD40 repeat region of WDR5 (WDR5_WD40_), the N-terminal domain of RbBP5 (RbBP5_NTD_) and full-length RbBP5 (which we refer to from here forward simply as RbBP5), followed by the characterization of dimeric and trimeric complexes. Figure 1A shows the protein constructs used in this study. Normalized Kratky plots of WDR5_WD40_ and RbBP5_NTD_ exhibit a typical bell-shape with a maximum at (1.73, 1.1) expected for globular proteins and are nearly superimposable in the q range 0<qRg<3 (Figure 1B). Also, the experimental values of Rg predicted for WDR5_WD40_ and RbBP5_NTD_ are in agreement with the theoretical values expected for globular proteins (Table 1 and Supplementary Figure S1). The normalized Kratky plot of MLL1_RBS-SET_ also exhibits a bell-shape, but its maximum is shifted with respect to the globular protein position, with poor convergence at high q-values, indicating that MLL1_RBS-SET_ contains flexible regions. The observed flexibility of MLL1_RBS-SET_ could be attributed to known inherent dynamics of the SET domain in the absence of cofactor (35), and to the disordered N-terminus of the MLL1_RBS-SET_ construct. The calculated solution ensembles for each protein taking into account known or predicted disordered regions (see Supplementary Data for details) establish good correspondence between our SAXS measurements and the crystal structures of WDR5 (53), the SET domain of MLL1 (45) and the WD40 domain of RbBP5 (54) (Supplementary Figure S2).

**Table 1.** SAXS parameters derived for the MLL1 trimeric complex, as well for its individual components and associated binary complexes

	Rg^a^ (Å)	Rg^b^ (Å)	D_max_^c^ (Å)	V_c_^d^	M_w_^e^	NSD^f^
*Individual Component*						
MLL1_WIN-SET_	20.2	20.8	73	207	22.0 (21.6)	0.58
WDR5_WD40_	19.8	19.9	70	251	25.9 (34.1)	0.60
RbBP5_NTD_	21.5	21.7	76	276	28.7 (36.8)	0.72
RbBP5	32.1	33.0	113	489	60.6 (59.1)	0.64
*Binary complexes*						
WDR5–MLL1_WIN-SET_	32.5	33.6	120	372	56.5 (64.7)	0.79
WDR5–RbBP5	39.8	41.1	140	669	91.2 (96.5)	0.79
*Trimeric complex*						
WDR5–RbBP5–MLL1_WIN-SET_	49.1	51.8	183	929	135.5 (124.6)	0.69

^a^Radius of gyration calculated using Guinier fit.

^b^Radius of gyration calculated using GNOM (60).

^c^Maximum distance between atoms calculated using GNOM.

^d^Volume of correlation (61).

^e^Molecular weight (M_w_) estimated from SAXS using V_c_ (61). The M_w_ expected from the sequence is shown in parentheses.

^f^NSD: Normalized spatial discrepancy; the values given are the average from fifteen runs of DAMMIF (62).

**Figure 2.**
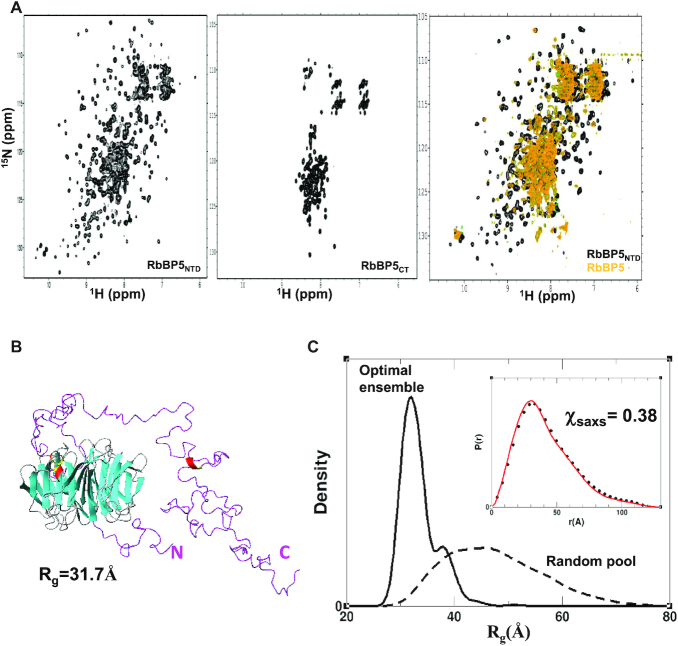
SAXS and NMR analysis of RbBP5. (**A**) (^1^H-^15^N)-TROSY spectra of the RbBP5_NTD_ β-propeller domain (left), RbBP5_CT_ (middle) and full-length RbBP5 (right). For the C-terminus (CT), the amide resonances are clustered between 7.5 and 8.5 ppm in the ^1^H dimension indicating that it is unfolded, while the well-dispersed spectrum of the N-terminal domain (NTD) is consistent with our homology model and the reported crystal structure (54) (PDB ID: 5OV3). Overlay of spectra of RbBP5_NTD_ with RbBP5 (right) shows that some β-propeller resonances exhibit peak shifts. (**B**) A representative member of the most populated model reflects a structured NTD and a flexible, but non-random CT. (**C**) The clear difference in Rg distribution profiles for the initial pool of 30 000 models (with random conformations of the CT (dashed)) versus the SAXS-derived ensemble (solid) indicate that the CT, in the context of full-length RbBP5, is not randomly disordered. Inset shows the pair distance distribution function P(r) calculated for the experimental data (black circles) and for the SAXS-derived ensemble (red line).

Initially, one of the main challenges in modeling the MLL1 complex was the lack of structural information on RbBP5. For our characterization and modeling of RbBP5-containing complexes we made use of a ROSETTA-derived homology model of its WD40 domain (i.e. RbBP5_NTD_). However, late in the course of manuscript preparation, Mittal *et al.* (54) reported the crystal structure of the mouse RbBP5 WD40 repeat region, which forms a canonical 7-unit β-propeller structure (PDB ID: 5OV3). The human and mouse WD40 domains of RbBP5 have 100% sequence identity and there is excellent agreement between our homology model and the reported structure (r.m.s.d. ∼2.1 Å; Supplementary Figure S2H), which we believe validates the model's use in our study. To help understand RbBP5 behavior in solution, we collected (^1^H-^15^N)-TROSY spectra of a full-length construct, as well as constructs corresponding to the C-terminus (CT) and NTD (Figure 2A). The spectrum of RbBP5_NTD_ is consistent with our model, and the reported β-propeller fold; there is considerable peak dispersion due to the high β-strand content, and we are able to identify ∼250 out of 316 expected backbone amide signals. We see a similar level of peak dispersion in TROSY spectra of WDR5_WD40_ (vide infra). We can distinguish approximately seven out of nine expected tryptophan indole signals based on their position in the lower left corner of the spectrum; however without resonance assignments, this cannot be unambiguously verified. Amide residues in long unstructured regions of proteins generally have poorly differentiated chemical environments and long relaxation times due to fast internal dynamics on the ps-ns timescale, resulting in sharp signals clustered between 7.5 and 8.5 ppm (55). The spectrum of RbBP5_CT_ indicates a lack of structure (Figure 2A). We are able to identify ∼120 peaks excluding putative side-chain signals that are expected to appear in the upper left region of the spectrum (i.e. 7.8-6.6 ppm for ^1^H and 115-110 ppm for ^15^N). The RbBP5_CT_ construct contains 199 residues of which 19 are prolines and no tryptophans, and it is likely that several peaks comprise signals from two or more amides. The TROSY spectrum of RbBP5 (Figure 2A and Supplementary Figure S3A–D) reflects features of both its folded and unfolded regions and is of poor quality, likely due to its large size and dynamic properties. Comparison of 1D-^1^H overlays corresponding to the first ^15^N increment of TROSY spectra of RbBP5, RbBP5_NTD_ and RbBP5_CT_ indicates that the strongest amide signals for RbBP5 are clustered in the center of the spectrum and arise from residues in the C-terminus (Supplementary Figure S3A). Nevertheless, several resonances from the β-propeller region are visible, and do not uniformly overlap with those in the spectrum of RbBP5_NTD_. The NMR spectra indicate that RbBP5 exhibits a high degree of disorder and this is consistent with its gel filtration profile (Supplementary Figure S3E). This is also reflected in its Rg, calculated from SAXS measurements (Table 1 and Supplementary Figure S1), and the shape of the normalized Rg-based Kratky plot and the pair distance distribution function P(r) (Figure 1B and C). In particular, the P(r) function has an asymmetric shape with a long smooth tail at large *r*-values, and the position of its maximum is shifted only slightly (∼4 Å) with respect to that of RbBP5_NTD_. The latter features indicate that RbBP5 has no globular content beyond its β-propeller domain. Moreover, sequence-based theoretical calculations of both secondary structure and order parameters also predict a rigid globular N-terminus and a flexible coil-like C-terminus (Supplementary Figure S3F). Molecular weight estimates derived from SAXS data indicate that both RbBP5 and RbBP5_NTD_ are monomeric in solution (Table 1).

We used the sparse ensemble selection (SES) approach (56) to calculate a solution ensemble of RbBP5 that would satisfy the SAXS data. An initial ensemble consisting of 20 000 models with random conformations of its flexible regions (i.e. residues 1–23 and 326–538) did not fit the SAXS data well (goodness-of-fit *χ_saxs_* = 9.4). We next generated an ensemble that better fits the SAXS data, by calculating an optimal weight for each model in the initial ensemble using a multi-orthogonal matching pursuit algorithm (56) (see Supplementary Data for details). The resulting optimal ensemble fits the SAXS data very well with *χ_saxs_* = 0.38 (Figure 2C and Supplementary Figure S2B)—the most populated models are shown in Figure 2B and Supplementary Figure S2F and G. In these models both N- and C-terminal regions preferably ‘fold in’, rather than adopt extended conformations (Supplementary Figure S2F). The optimal ensemble displays a much more narrow Rg distribution than the initial random ensemble, with a major peak at 37 Å (Figure 2C). This indicates that RbBP5 is more compact than would be predicted if its C-terminus was fully random.

### Binary subcomplexes have dynamic non-random solution conformations mediated by WD40 repeat domains

**Figure 3.**
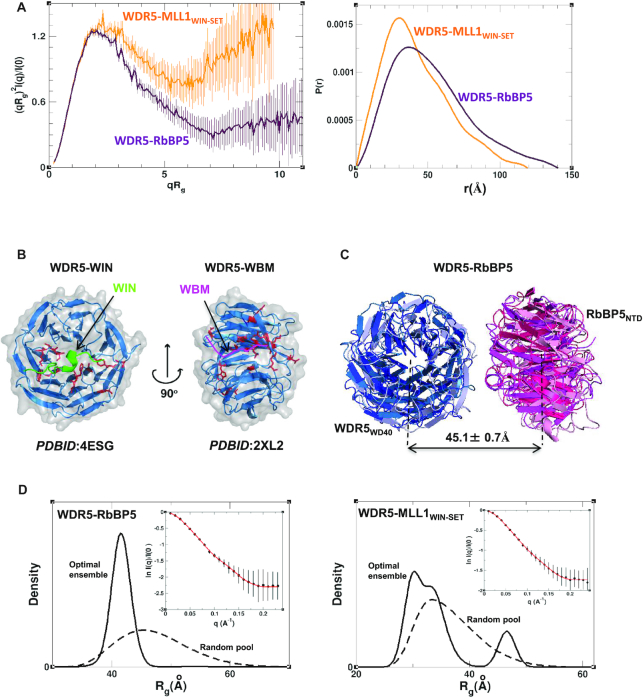
WDR5–MLL1 and WDR5–RbBP5 binary complexes. (**A**) Rg-based Kratky plots (left) of SAXS data for binary complexes of WDR5–MLL1_WIN-SET_ (orange) and WDR5–RbBP5 (maroon). Normalized pair distance distribution functions P(r) calculated from experimental SAXS data with GNOM (right). The data indicate that while both complexes are flexible, WDR5–MLL1_WIN-SET_ possesses a significantly higher degree of flexibility than WDR5–RbBP5. (**B**) WDR5 residues (red, in stick representation) that exhibit the highest CSPs in (^1^H-^15^N)-TROSY titrations with peptides corresponding to the WIN (green) and WBM motifs (pink) (CSPs > 0.15 ppm for WIN peptide, CSPs > 0.23 for WBM peptide). CSPs are mapped onto the crystal structures of WDR5–WIN peptide (PDB ID: 4ESG) and WDR5–WBM peptide (PDB ID: 2XL2). Histograms presenting complete CSP data obtained from the two titrations are presented in Supplementary Figure S5A. (**C**) The centers of geometry of RbBP5_NTD_ (pink) and WDR5_WD40_ (blue) domains are positioned at about the same distance (∼45 Å) in 95% of the models in the optimal ensemble. (**D**) Rg distribution for the initial pool of random structures (dashed) and for the SAXS-derived optimal ensemble (solid) of WDR5–RbBP5 (left) and WDR5–MLL1_WIN-SET_ (right). Experimental SAXS profiles (black circles) with theoretical profiles (red line) averaged over the SES ensemble (inset).

Our SAXS data for the binary complexes of WDR5–MLL1_WIN-SET_ and WDR5–RbBP5 both suggest the presence of significant disorder, especially for WDR5–MLL1_WIN-SET_ (Figure 3A). The P(r) functions of WDR5–MLL1_WIN-SET_ and WDR5–RbBP5 are typical for proteins containing globular domains tethered by long disordered regions (Figure 3A). The position of the P(r) major peaks for the aforementioned complexes is close to their respective positions for the individual components (Figure 1C), indicating that in both complexes the globular domains are not in close contact and may not adopt a unique arrangement in solution. WDR5 is known to interact with RbBP5 and MLL1 through small peptide segments designated as the WDR5 binding motif (WBM) (43) and WDR5 interacting (WIN) motif (41), respectively (Figure 1A). Both interactions have reported dissociation constants on the order of 1–2 μM (36,41,43,44). To calculate solution ensembles of the binary complexes, we first used (^1^H-^15^N)-TROSY titrations to verify that WDR5′s interaction with the motifs, as observed in the crystal structures, is maintained in solution. To this end, we expressed a triply labeled (^15^N/^13^C/^2^H) WDR5_WD40_ construct (which contains 311 residues) and assigned 254 backbone spin systems representing 82% of the sequence (Supplementary Figure S4). The assignments have been deposited in the BMRB database (BMRB_ID: 27528). Amide resonance chemical shift perturbations (CSPs) were then quantified for WDR5 titrated with peptides corresponding to the two motifs. Residues with the highest CSPs (Supplementary Figure S5A) were mapped onto the WIN (PDB ID: 4ESG) and WBM (PDB ID: 2XL2) peptide-bound crystal structures (Figure 3B). For both titrations, all of the assigned WDR5 residues at the binding interface were among those with the highest CSPs (Supplementary Figure S5A). We are therefore confident in using the crystal structures to delineate restraints governing the interaction of these motifs with WDR5 in our modeling of the binary complexes.

The binary subcomplexes, which are both flexible, exhibit different structural organizations. The optimal ensemble for WDR5–MLL1_WIN-SET_ has an Rg distribution as broad as the initial random ensemble (Figure 3D), and the arrangement of the globular domains in the most populated models does not support the existence of additional interactions outside of the WIN motif (Supplementary Figure S5). In contrast, the optimal ensemble for the WDR5–RbBP5 displays a relatively narrow Rg distribution, with a major peak at ∼41 Å (Figure 3D), indicating the predominantly populated conformations are more compact than those in the initial random ensemble. The relative position of the WDR5 and RbBP5 WD40 domains in the ensemble are well defined, with a distance between their centres of mass (*d_WR_*) equal to 45.1 ± 0.7 Å (Figure 3C and Supplementary S5G). There is no apparent direct contact between the domains and their relative orientation with respect to each other is variable. The r.m.s.d. between highly populated conformers in the optimal ensemble is ∼18 Å due to the interdomain dynamics. The preference for compact conformers may be explained by the formation of interactions between RbBP5_CT_ and the two β-propeller domains. These contacts cannot be more precisely defined due to our use of rigid-body models in the calculations. We used biolayer interferometry (BLI) to estimate the binding affinity for WDR5–RbBP5 interaction, and in our hands found the K_D_ to be ∼ 0.3 μM (Supplementary Figure S5H and Table S1). This compares to a value of ∼2.4 μM estimated using analytical ultracentrifugation by Cosgrove and colleagues (31) (Supplementary Table S1).

In summary, our structural analysis of the binary subcomplexes suggests that WDR5–RbBP5 is relatively compact, with a well-defined distance between the WD40 domains. In contrast, WDR5–MLL1_WIN-SET_ has a significantly higher degree of flexibility, with a broad interdomain distance distribution profile. It should be noted that SAXS measurements were collected for a putative RbBP5–MLL1_RBS-SET_ complex, however the data were not of high enough quality to proceed with structural analysis. We believe this is due to the weak affinity between MLL1_RBS-SET_ and RbBP5, as compared to the intermolecular affinities observed with the WDR5–RbBP5 and WDR5–MLL_WIN-SET_ pairs (see Supplementary Table S1).

### SAXS and cross-linking data suggest a dynamic triangulated ensemble for WDR5–RbBP5–MLL1_WIN-SET_

**Figure 4.**
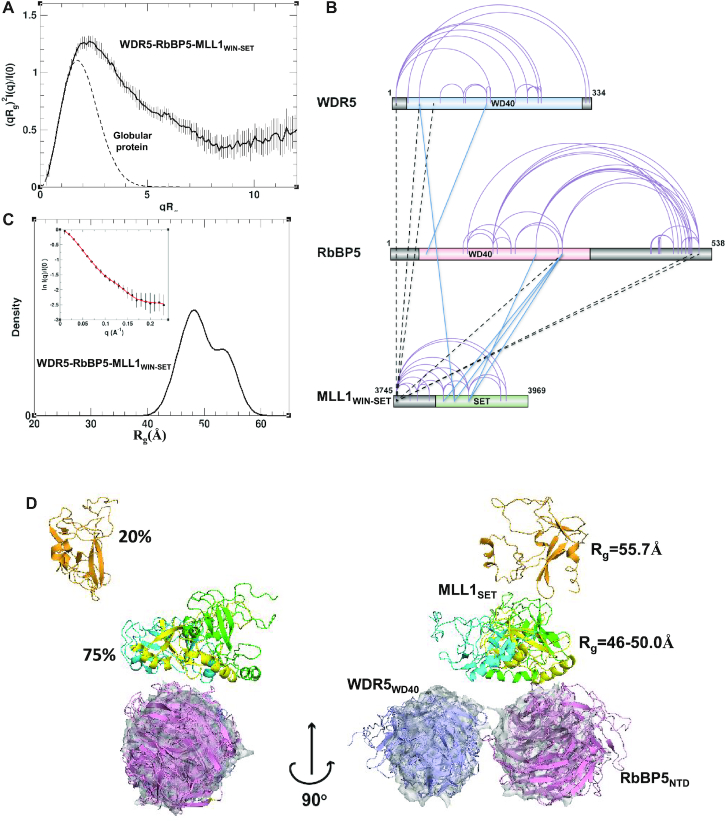
Dynamic model of the trimeric WDR5–RbBP5–MLL1_WIN-SET_ complex derived from SAXS and cross-link data. (**A**) Rg-based Kratky plot of SAXS data for WDR5–RbBP5–MLL1_WIN-SET_ indicates a high degree of flexibility. (**B**) Sequence mapping of intraprotein and interprotein cross-links. Intraprotein cross-links are indicated with purple arcs, while interprotein cross-links between globular domains or flexible regions are indicated with blue and dashed-black lines, respectively. (**C**) Rg distribution for the optimal ensemble of the trimer is shown by a solid black line. Experimental SAXS profile (black circles) plotted with theoretical profiles (red line) averaged over the ensemble (inset). (**D**) Cartoon diagram of the four most populated models of the optimal ensemble. Only structured domains are shown. The WD40 domains of RbBP5 and WDR5 are colored in pink and blue, respectively, while MLL1 SET domain is colored differently in each model (green, cyan, yellow and orange). For reference, the WD40 domains of each model are superimposed with the most populated model of the WDR5–RbBP5 dimer (Figure 3) displayed in a semi-transparent surface representation (gray).

Our SAXS data for the WDR5–RbBP5–MLL1_WIN-SET_ complex showed significant flexibility in the sample. The shape of the experimental Kratky plots of the complex is typical of proteins with substantial interdomain flexibility (Figure 4A and Supplementary Figure S6A). In particular, the Rg-based Kratky plot is a bell-shaped curve with a maximum at (2.26, 1.27), coordinates which are shifted to higher values with respect to those expected for a globular protein. Also, the presence of a high degree of flexibility is evidenced by the poor convergence of the Kratky plots at high *q*-values. The low maximum value of 0.48 in the Vc-based Kratky plot (Supplementary Figure S6A), as well as the asymmetric shape of the P(r) function (Supplementary Figure S6B), suggests an elongated shape. This agrees with the averaged *ab initio* SAXS-derived molecular envelope, which showed an extended shape with approximate dimensions of 220 × 105 × 70 Å (Supplementary Figure S6C).

We note that pair distance distribution functions of proteins containing several globular domains, connected by long disordered regions, are characterized by peaks at low *r*-values, corresponding to intradomain distances. Therefore, if the three globular domains of WDR5, MLL1_WIN-SET_ and RbBP5 are not interacting directly with each other within the trimer, we would expect the P(r) function to have peaks at 26–32 Å, reflecting the interatomic distances prevailing within these domains (Figure 1C and Supplementary Figure S6B). However, the experimental P(r) function has its maximum at a much larger distance of ∼47 Å (Supplementary Figure S6B), suggesting the existence of interdomain contacts.

To aid our modeling of the trimeric complex, we performed XL-MS studies. We observed many intramolecular cross-links within each of the three proteins. These were highly consistent with the available WDR5 (53), MLL1_WIN-SET_ (45) and RbBP5_NTD_ (54) crystal structures indicating that the models are reliable representations of the domains within the complex in solution. We also observed a number of intermolecular cross-links, with the largest number being between MLL1 and RbBP5 suggesting they are in close proximity. Figure 4B shows sequence mapping of both intra- and intermolecular DSS cross-links. There are six intermolecular cross-links between lysine residues within the globular subunits that are shown on Figure 4B by solid blue lines. All 31 experimentally observed cross-links were used in the modeling (Supplementary Tables S2 and 3).

Using both SAXS and cross-linking data as conformational restraints, we utilized the SES approach to calculate solution ensembles of WDR5–RbBP5–MLL1_WIN-SET_ that satisfy both sets of experimental data. An initial pool of representative structures was generated by combining rigid-body modeling and molecular dynamics simulations for both all-atomic and coarse-grained models along with cross-link derived distance restraints (see Supplementary Data for details). It was assumed that MLL1_WIN-SET_ and RbBP5 were tethered to WDR5 via the WIN and WBM motifs, respectively, as seen in crystal structures (36,41–43). It should be noted here that individual members of the initial ensemble of conformers did not necessary satisfy all intermolecular cross-links: each satisfied on average three to four.

The optimal ensemble of WDR5–RbBP5–MLL1_WIN-SET_ fits the SAXS data as a whole, with *χ*_*saxs*_ = 0.23 in the *q*-range 0<q<0.23. While only SAXS data were used to select the optimal ensemble, each experimentally observed cross-link is consistent with at least one member, so that the ensemble as a whole is consistent with all 31 cross-links. The expected Rg exhibits a wide distribution with a maximum at ∼48 Å (Figure 4C) and the SES-derived ensemble suggests that the complex can assume a range of interdomain arrangements in solution (Figure 4 and Supplementary Figure S6C). One notable feature of the optimal ensemble is that the β-propeller domains of WDR5 and RbBP5 adopt the same relative positions within the trimer as they do in the WDR5–RbBP5 dimer (Figure 4D and Supplementary Figure S6E). The placement of the MLL1 SET domain is more variable. Most conformers (∼80%) adopt a compact arrangement in which the SET domain, and the two WD40 domains are in close proximity (Figure 4D). However, in a small population of conformers (∼20%), the SET domain is ‘detached’. When the conformers adopt a compact conformation, the relative position of the SET and RbBP5 β-propeller domains is well-defined (Supplementary Figure S6F) and this is supported by four interdomain cross-links (Figure 4B; Supplementary Figure S6D and Table S2). However, WDR5′s positioning varies because its contact with RbBP5 and MLL1 occurs within their flexible linker regions. There are only two interdomain cross-links that involve WDR5, and they can only be satisfied simultaneously in ∼10% of the conformers of the optimal ensemble.

### The WD40 β-propeller domain of RbBP5 has a unique interaction with MLL1

A crystallographic study by Li *et al.* (35) highlighted the critical role of the AS+ABM region of RbBP5 in stimulating SET domain methyltransferase activity in the MLL family. The catalytic activities of MLL2/3/4 were found to be highly dependent on the presence of RbBP5_AS+ABM_-ASH2L_SPRY_. In contrast, methyltransferase activity of MLL1 was weakly stimulated by RbBP5_AS+ABM_-ASH2L_SPRY_, and more dependent on WDR5. The authors identified a surface of the SET domain (in the I-SET motif) that serves as a hub for MLL–RbBP5–ASH2L interaction. Two MLL1 residues at this surface (Asn3861 and Gln3867) have different side-chain properties compared to MLL2/3/4 (hydrophilic/bulky *vs*. hydrophobic) that prevent ‘optimal’ RbBP5_AS+ABM_-ASH2L_SPRY_ interaction. Mutation of these two residues to their MLL2 (or MLL3) counterparts restored the binding interface, such that MLL1 could be crystallized with (PDB ID: 5F6L) and its methyltransferase activity stimulated by the RbBP5–ASH2L dimer.

**Figure 5.**
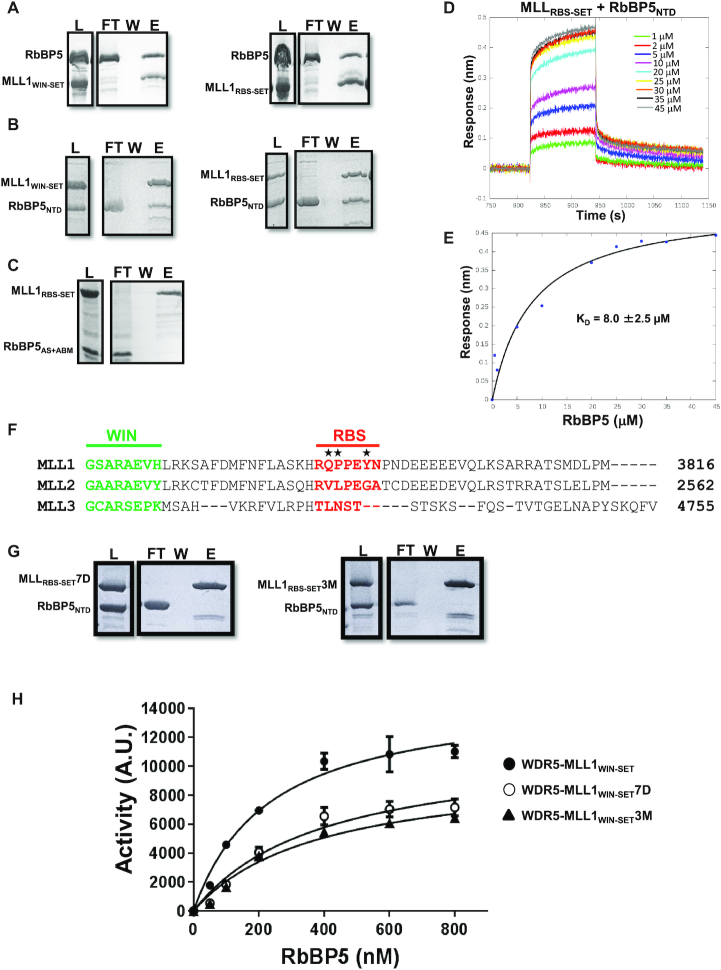
The N-terminal β-propeller domain of RbBP5 binds to MLL1. GST pull-down experiments (where MLL constructs are GST-tagged) show direct interaction between (**A**) RbBP5 and (**B**) RbBP5_NTD_ with MLL1_WIN-SET_ and MLL1_RBS-SET_, (**C**) whereas no interaction is observed between RbBP5_AS+ABM_ and MLL1. Control experiments were performed to show that the GST tag was not involved in the interaction (data not shown). (**D**) BLI sensorgrams for the binding of RbBP5_NTD_ to sensor-immobilized MLL1_RBS-SET_, (**E**) and the corresponding steady-state binding curve. (**F**) Sequence alignment for MLL1, MLL2 and MLL3 linker residues between the WIN motif and SET domain, with the WIN motif in green and RBS in red. (**G**) In GST pull-down experiments (where MLL1 constructs are GST-tagged), no interaction is observed between RbBP5_NTD_ and MLL1_RBS-SET_7D (i.e. the entire RBS is deleted) or MLL1_RBS-SET_3M (i.e. Q3787V, P3788L, Y3791G, MLL1 to MLL2 mutation—marked above with stars in panel F). (**H**) In histone methyltransferase activity assays, MLL1_WIN-SET_7D and MLL1_WIN-SET_3M constructs have significantly reduced catalytic activity compared to wild-type MLL1_WIN-SET_. (For pull-down experiments: L = Load, FT = Flow-through, W = Wash, E = Eluate)

Our study of the WDR5–RbBP5–MLL1_WIN-SET_ complex provides a basis for understanding how MLL1 methyltransferase activity is stimulated by RbBP5 and WDR5. When the trimer adopts a compact configuration (found in ∼75% of the optimal ensemble conformers), we observe a direct interaction between the WD40 domain of RbBP5 and a short peptide sequence of MLL1 located between its WIN motif and SET domain. We refer to this 7-residue RbBP5 binding sequence as the RBS region of MLL1 (Figure 1A). To confirm this specific interaction, we performed GST pull-down and BLI binding studies with several RbBP5 and GST-tagged MLL constructs (Figure 5). Both MLL1_RBS-SET_ and MLL1_WIN-SET_ were found to interact exclusively with RbBP5 constructs containing the N-terminal WD40 domain (Figure 5A and B). No interaction is observed between MLL1 and RbBP5 AS-containing C-terminal constructs in GST pull-down (Figure 5C) or BLI assays (data not shown). BLI was used to estimate the *K*_D_ for the interaction of MLL1_RBS-SET_ and RbBP5_NTD_, and found to be ∼8 μM (Figure 5D and E).

The RBS is a unique feature of MLL1 and is not conserved in MLL2/3/4 which rely strongly on the ASH2L-RbBP5 dimer for activation (Figure 5F). We performed mutagenesis experiments to confirm the importance of the RBS in promoting MLL1 interaction with RbBP5, and in stimulating the methyltransferase activity of the SET domain. We constructed two MLL1 mutants: the first, MLL_WIN-SET_7D, where its 7-residue RBS was deleted; and the second, MLL1_WIN-SET_3M, where three mutations (Q3787V, P3788L and Y3791G) transform MLL1 RBS to the corresponding sequence of MLL2. Both mutants failed to bind to RbBP5 constructs containing the N-terminal WD40 domain (Figure 5G). Furthermore, we found that both deletion or mutation of MLL1 RBS decreased RbBP5′s ability to stimulate methyltransferase activity of the WDR5–MLL1_WIN-SET_ complex (Figure 5H).

**Figure 6.**
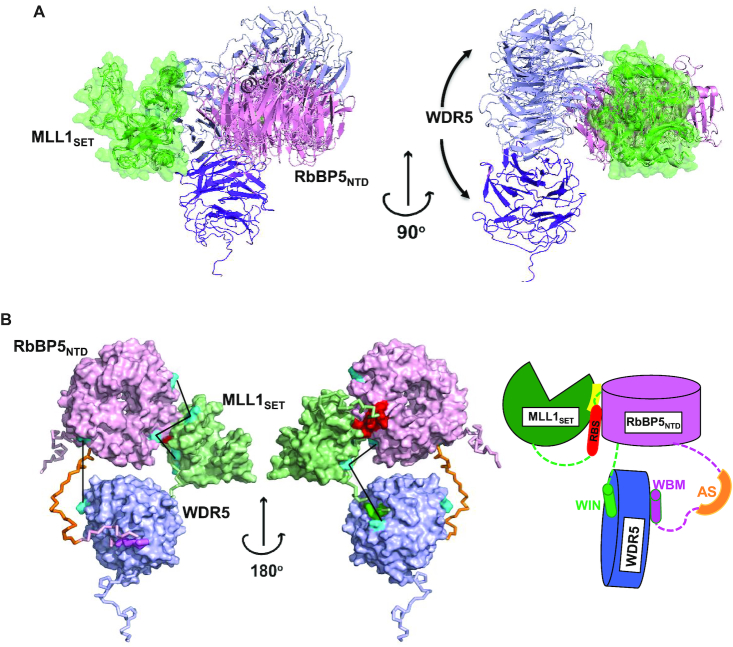
Organization of the WDR5–RbBP5–MLL1_WIN-SET_ trimer. (**A**) Cartoon diagram of models representing the major population (∼80%) of the optimal ensemble. Only structured WDR5_WD40_ (purple/blue), RbBP5_NTD_ (pink) and MLL1_SET_ (green) domains are shown. For different members of the ensemble, the SET domain is superimposed and shown by both cartoon and transparent surface representation. (**B**) The most populated conformer representing the MLL1 trimer is shown by a surface representation of the globular regions and a backbone trace for the flexible ones. For clarity, the CT of RbBP5 (i.e. RbBP5_382-538_) is not shown and the residues from the AS+ABM region are in dark orange, WIN in green, WBM in violet and RBS in red. The cross-links between globular domains are shown by solid black lines and the cross-linked lysine residues are shown in cyan. Schematic representation of the domain arrangement and critical motif and segment interactions seen in the model are shown to the right.

Our primary model for WDR5–RbBP5–MLL1_WIN-SET_, which represents the majority of compact conformers in our optimal ensemble is presented in Figure 6. In this model, only the β-propeller domain of RbBP5 makes contact with the MLL1 SET domain—the AS is not positioned correctly to enable contact with the SET domain. The RBS binding surface of RbBP5_NTD_ consists of a number of hydrophobic residues (V249, W279, I283, L286, V287 and I289), as well as Q273, Y277 and P253 (Supplementary Figure S6G). The RBS may also participate in an intramolecular association with the SET domain which serves to bridge the RbBP5/SET interaction. Interestingly, in our model we see an interaction between RbBP5_NTD_ and Asn3861 of the SET domain, which as noted above, was identified as one of the two critical residues that distinguishes MLL1 from MLL2/3/4 *vis-à-vis* its ability to interact with RbBP5–ASH2L. SET domain residues that form the putative RBS + RbBP5_NTD_ binding interface (K3825, K3828, N3861, R3871, M3897, H3898, G3899, R3903 and F3904) are shown in Supplementary Figure S6H.

Within our compact trimer optimal ensemble (Figure 4D) we see a small population of conformers that adopt a domain arrangement where the RbBP5 AS is favorably positioned to interact with the SET domain (Supplementary Figure S7). It is important to note that in these species, the RBS maintains its contact with RbBP5 NTD as seen in our primary model, however it no longer forms the intramolecular bridge with the SET domain (Figure 6 and Supplementary Figure S7). This minor population of conformers highlights the potential for dual NTD/AS RbBP5 contacts with MLL1. Our GST pull-down and BLI binding studies show that the AS does not on its own, measurably interact with the SET domain (Figure 5). However, it is possible there could be an avidity effect, where RBS binding to RbBP5_NTD_ promotes SET domain/AS interaction. Our attempts to confirm this avidity under multiple buffer conditions using BLI were inconclusive—while we see potentially stronger SET domain binding using RbBP5 constructs having both the NTD and AS+ABM region (Supplementary Figure S8), the binding behavior gave rise to non-ideal BLI sensorgrams, without steady-state and complete dissociation phases needed for proper *K*_D_ determination. We believe this is due to protein aggregation in the assays. At present, we can only speculate that within the context of the trimer, the presence of full-length RbBP5 and WDR5 may facilitate some level of AS/SET interaction. This is supported by MD simulations of the all-atom model of the trimer which were initiated with the three globular domains positioned according to the minor population in the ensemble (Supplementary Figure S7), and with the AS positioned in contact with MLL1 SET as per the crystal structure of MLL1_N3861I/Q3867L_ bound to the RbBP5_AS+ABM_-ASH2L_SPRY_ dimer (32). Throughout the course of the MD trajectory (100 ns), the AS maintained constant contact with the SET domain.

**Figure 7.**
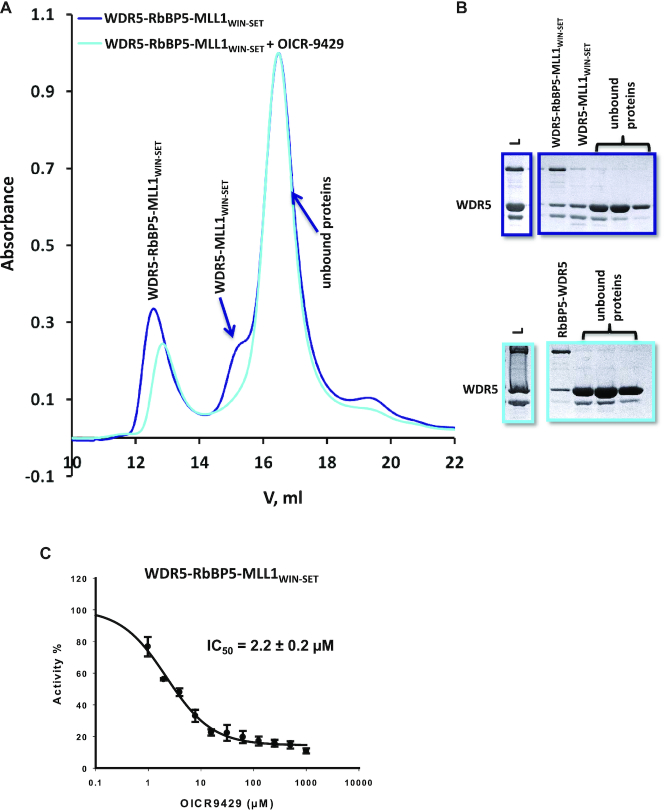
OICR-9429 attenuates the assembly of a functional trimeric complex. (**A**) Size exclusion chromatography of WDR5–RbBP5–MLL1_WIN-SET_ in the absence (navy) or presence of (cyan) of ∼5-fold molar excess OICR-9429. This compound binds to WDR5 (*K*_D_ = 93 nM) (38) and competes with the MLL1 WIN motif. (**B**) SDS-PAGE of elution fractions (L = Load). Fractions containing the trimer and WDR5–MLL1_WIN-SET_ (shoulder at 15.2 ml) are not recovered from the column when run in the presence of OICR-9429. (**C**) OICR-9429 inhibits the catalytic activity of the trimer.

Taken together, our structural characterization of WDR5–RbBP5–MLL1_WIN-SET_ suggests that its activation is mediated in part through the unique, but weak interaction of the MLL1 RBS with RbBP5, which in turn stabilizes the SET-I motif of the catalytic SET domain. WDR5 serves as a hub to promote this interaction, through its dual binding to the WIN (on MLL1) and WBM motifs (on RbBP5). Hence, we hypothesize that a triumvirate of weak, but specific intermolecular interactions are required to maintain the integrity of the MLL1 minimal complex, and that disruption of an individual interaction site may be sufficient to disrupt catalytic activity. To test this hypothesis, we measured the ability of OICR-9429, a small molecule antagonist of WDR5–MLL1_WIN_ interaction, to disrupt the association of WDR5–RbBP5–MLL1_WIN-SET_ using gel filtration (Figure 7A). The disruption of WDR5–MLL1 interaction by the compound compromised the assembly of the trimer (Figure 7A and B), and inhibited its catalytic activity (Figure 7C). This is consistent with our previous work showing that OICR-9429 can disrupt the assembly and function of endogenous MLL1 complexes in cells (38). Similar results have been reported for MM-401, a peptide-based antagonist of WDR5–MLL_WIN_ interaction (39,57). This has important implications for the development of pharmacological antagonists of the MLL1 complex, and further strengthens this approach to target other multiprotein complexes that are dependent on weak, but druggable interactions.

During revision of our manuscript, the crystal (58) (PDB ID: 6CHG) and cryo-EM structures (59) (PDB ID: 6BX3) for the yeast COMPASS were reported, which comprises orthologues of SET1, WDR5, RbBP5, ASH2L and DPY30. It is interesting to note that the relative position of the WD40 domains of WDR5 and RbBP5 is conserved not only in our dimer and trimer models, but is also consistent with the orientation in the reported COMPASS structures (Supplementary Figure S9A). However, the relative positions of the SET and two WD40 domains adopted in COMPASS (Supplementary Figure S9B) is not consistent with our experimental SAXS and cross-links data obtained for the MLL1 trimer (Supplementary Figure S9C). Moreover, the domain arrangement in COMPASS is not compatible with any of the conformers that make up our optimal ensemble of the minimal MLL1 trimer. This extends as well to our preliminary characterization of SAXS and cross-links data for the MLL1 pentameric complex. These differences suggest additional evidence of the distinct properties of MLL1 among the SET1 family of enzymes.

## Supplementary Material

gkz697_Supplemental_Files
