# Supplementary material for: The MLL1 trimeric catalytic complex is a dynamic conformational ensemble stabilized by multiple weak interactions
Source: Nucleic Acids Res. 2019 Aug 10;47(17):9433–47. doi: 10.1093/nar/gkz697 (PMC6755125; doi:10.1093/nar/gkz697)
Supplement: gkz697_Supplemental_Files [file gkz697_supplemental_files.zip › MLL_complex_Supplementary_Data_july22.pdf]

Supplementary Data for:

The MLL1 trimeric catalytic complex is a dynamic conformational ensemble stabilized by multiple weak interactions

Lilia Kaustov, Alexander Lemak, Hong Wu, Marco Faini, Lixin Fan, Xianyang Fang, Hong Zeng, Shili Duan, Abdellah Allali-Hassani, Fengling Li, Yong Wei, Masoud Vedadi, Ruedi Aebersold, Yunxing Wang, Scott Houliston, Cheryl H. Arrowsmith

Detailed Materials and Methods:

Protein expression and purification.

Human MLL1 constructs containing residues 3745-3969 ($MLL1_{WIN\text{-}SET}$) and 3785-3969 ($MLL1_{RBS\text{-}SET}$), as well as the WD40 repeat region of WDR5 (residues 24-334), the N- and C-terminus of RbBP5 ($RbBP5_{NTD}$, $RbBP5_{CT}$), and full-length RbBP5 were individually expressed in *E. Coli* (BL21(DE3) codon plus RIL, Agilent). The MLL1 proteins were expressed as N-terminal GST fusions and purified on a GST-bind (Novagen) column according to manufacturer's instruction. The GST tag was cleaved off by incubating the resin-bound fusion protein with thrombin (Sigma) at 4°C overnight. The eluted MLL1 proteins were passed through a gel filtration column (Superdex 200, GE Healthcare) pre-equilibrated with 20 mM Tris-HCl (pH 7.5), 500 mM NaCl. WDR5 and RbBP5 proteins were expressed as N-terminal Hexa-His fusions and purified on Nickle-chelating column (GE Healthcare). After elution and removing His-tag by incubation with TEV protease at 4°C overnight, the proteins were subjected to a gel filtration column Superdex 200 (GE Healthcare) pre-equilibrated with 20 mM Tris-HCl (pH 8.0), 500 mM NaCl (for RbBP5) and 20 mM PIPES (pH 6.5), 250 mM NaCl (for WDR5), respectively.

The dimeric and trimeric complexes of MLL1 used for SAXS and cross-linking studies were expressed in Sf9 cells. The dimeric complex of WDR5-$MLL1_{WIN\text{-}SET}$ and WDR5-RbBP5 were purified by TALON affinity column (Clontech) followed by a gel filtration column (Superdex 200, GE Healthcare) pre-equilibrated with 20 mM BisTris propane (pH 7.0), 250 mM NaCl. The fractions containing the two dimeric complexes were collected separately and used for SAXS data collection. The two dimeric complexes were mixed and incubated on ice for 2 hours and further purified on a gel filtration column (Superdex 200, GE Healthcare) pre-equilibrated with 20 mM BisTris propane (pH 7.0), 250 mM NaCl. The fractions containing the trimeric complex were collected and used for SAXS data collection and cross-linking experiments.

SAXS data collection and analysis

SAXS measurements were carried out at the beamline 12-ID-B of the Advanced Photon Source, Argonne National Laboratory. The energy of the X-ray beam was 14 Kev (wavelength λ=0. 8856 Å), and two setups (small- and wide- angle X-ray scattering, SAXS and WAXS) were

used simultaneously in which the sample to Pilatus 2M detector distance were adjusted to achieve scattering q values of $0.006 < q < 2.6 Å^{-1}$, where $q = (4\pi/\lambda)\sin\theta$, and 2θ is the scattering angle. Thirty two-dimensional images were recorded for each buffer or sample solutions using a flow cell, with accumulated exposure time of 0.8-2 seconds to reduce radiation damage and obtain good statistics. No radiation damage was observed as confirmed by the absence of systematic signal changes in sequentially collected X-ray scattering images. The 2D images were corrected and reduced to 1D scattering profiles using the Matlab software package at the beamlines. The 1D SAXS profiles were grouped by sample and averaged. The scattering profile of the protein was calculated by subtracting the background buffer contribution from the sample-buffer profile using the program PRIMUS (ATSAS package, EMBL) (1). Concentration series measurements for each sample were carried out to remove the scattering contribution due to interparticle interactions and to extrapolate the data to infinite dilution. The protein concentration ranges used for WDR5-MLL1$_{WIN-SET}$, RbBP5-WDR5, and WDR5-RbBP5-MLL1$_{WIN-SET}$ were 14-28 μM, 10-36 μM and 9.6-46.5 μM, respectively. Guinier analysis and the experimental radius of gyration (R_g) estimation from the data of infinite dilution were performed using PRIMUS. The pair distance distribution function (PDDF), P(r), and the maximum dimension of the protein, D_{max}, in real space was calculated with the indirect Fourier transform using the program GNOM (2). To avoid under-estimation of the molecular dimension and consequent distortion in low resolution structural reconstruction, the parameter D_{max}, the upper end of distance r, was chosen such that the resulting PDDF has a short, near zero-value tail at large r. The Rg from P(r) analysis was also reported. The Volume of correlation (3), V_c, was calculated using an in-house script. The molecular weights were estimated using V_c (3) in the q range of $0 < q < 0.3$ $Å^{-1}$. Fifteen *ab-initio* shape reconstructions (molecular envelopes) were generated using DAMMIF (4) and averaged with DAMAVER (5). The structural models were superimposed and overlaid with the averaged envelope using SUPCOMP (6). The theoretical scattering intensity of a structural model was calculated and fitted to the experimental scattering intensity using CRYSOL (7) and FoXS (8) programs.

Chemical cross-linking mass spectrometry

The reconstituted trimer complex of WDR5, RbBP5 and MLL1$_{WIN-SET}$ was cross-linked at a concentration between 12 and 16 μM with 1 mM of isotopically coded disuccinimidyl suberate

($DSS-d_0$,$DSS-d_{12}$) for 30 minutes at 37 °C while shaking at 500 rpm on a Thermomixer (Eppendorf) as previously described (9). Samples were quenched with 50 mM NH_4HCO_3 for 20 minutes at 37°C and evaporated to dryness in a vacuum centrifuge. The dried pellets were then dissolved in 50 µL of 8 M urea, reduced with 2.5 mM Tris(2-carboxyethyl)phosphine (Pierce) for 30 minutes at 37°C and alkylated with 5 mM iodoacetamide (Sigma-Aldrich) for 30 minutes at room temperature, in the dark. Digestion was carried out after diluting urea to 5 M with 50 mM NH_4HCO_3 and adding 1% (w/w) LysC protease (Wako Chemicals) for 2 hours at 37°C and subsequently diluting to 1 M urea with 50 mM NH_4HCO_3 and further adding 2% (w/w) trypsin (Promega) for 14 hours at 37°C. The digestion was stopped by acidification with 1% (v/v) formic acid. Digested peptides were purified using Sep-Pak C18 cartridges (Waters) according to the manufacturer's protocol. Cross-linked peptides were enriched by peptide size-exclusion chromatography (SEC) as previously described (9). SEC fractions were then reconstituted in 5% acetonitrile and 0.1% formic acid and analysed in duplicates on a LC (Easy-nLC 300) coupled to a mass spectrometer (Orbitrap LTQ XL). Analytes were separated on self-packed New Objective PicoFrit columns (11 cm x 0.075 mm I.D.) containing Magic C18 material (Michrom, 3 µm particle size, 200 Å pore size) over a 60-min gradient from 7% to 35% acetonitrile at a flow rate of 300 nL/min. The mass spectrometer was operated in data-dependent acquisition (DDA) mode with MS acquisition in the Orbitrap analyzer at 60,000 resolution and MS/MS acquisition in the linear ion trap at normal resolution after collision-induced dissociation. DDA was set up to select up to five most abundant precursors with a charge state of +3 or higher (9). MS data were converted to mzXML format with msConvert (10) and searched with xQuest/xProphet (11) against a database containing the FASTA sequences of the analysed proteins and relative decoy sequences. Cross-linked peptides were identified with a minimal length of 5 amino acids and at least four bond cleavages or three adjacent ones per peptide. Validated cross-linked peptides had a total ion current higher that 0.1 and xQuest score higher than 20. Figures were prepared with xiNET (12).

Structural characterization using SAXS data

The SAXS data indicate that the trimeric complex and its sub-complexes, as well as individual molecules $MLL1_{RBS-SET}$ and RbBP5, are flexible molecular systems in solution. Thus we took an ensemble approach for the characterization of these systems by utilizing the SES

protocol (13). The strategy on which the SES method is based consists of two main steps: 1) generate the initial ensemble of conformations in order to approximate the conformational space available for a system in solution; 2) find optimal weight w_k for each conformation k from the initial ensemble that minimizes the discrepancy:

$$\chi^2 = \sum_{i=1}^{N_q} \left[\frac{\sum_{k=1}^{N_{ens}} I_{calc}^k(q_i) \cdot w_k - I_{\exp}(q_i)}{\sigma(q_i)} \right]^2$$

where $I_{exp}(q)$ is the experimental scattering intensity, N_q is number of experimental points, $\sigma(q)$ is the experimental error, and $I_{calc}^k(q)$ is scattering intensity predicted for the kth conformation, and N_{ens} is the number of conformations in the initial ensemble. Multi-orthogonal matching pursuit (13) is used to find possible ensembles on step 2, and the optimal ensemble size was selected using the l-curve. The optimal weights were then obtained by w_k averaging over top solutions with similar χ^2.

The quality of the fit between the experimental data and those predicted from the ensemble of models is described by the following fitting parameter:

$$\chi_{SAXS} = \left[\frac{1}{N_q} \sum_{i=1}^{N_q} \left[\frac{I_{exp}(q_i) - \alpha \cdot I_{calc}^{ens}(q_i)}{\sigma(q_i)} \right]^2 \right]^{1/2}$$

where,

$$\alpha = \sum_{i=1}^{N_q} I_{exp}(q_i) \cdot I_{calc}^{ens}(q_i) / \sum_{i=1}^{N_q} I_{exp}(q_i) \cdot I_{exp}(q_i),$$

and,

$$I_{calc}^{ens}(q_i) = \sum_{k=1}^{N_{ens}} I_{calc}^{k}(q_i) \cdot w_k$$

The distribution of Rg and Dmax in an ensemble were obtained by the kernel density estimate using a Gaussian kernel with the bandwidth set to 1.5 Å and 5 Å, respectively. The distribution of these global parameters indicate the most populated conformations in solution. Comparison of

the distribution of an optimal ensemble relative to the distribution of the initial pool provides an indication of the degree of compactness or the extended nature of the system.

Generation of the structural ensembles

MLL1$_{RBS-SET}$: The high degree of flexibility observed for the MLL1$_{\text{RBS-SET}}$ sample originates from inherent flexibility of the SET domain and the 28 residue disordered N-terminal tail. We used all-atom molecular dynamics simulations to generate an initial ensemble of conformers. We then used all-atom MD simulations to generate a trajectory starting from the known crystal structure of the MLL1 SET domain with the cofactor product AdoHcy (14) (*PDBID*: 2W5Y). After minimization and equilibration, a productive run was continued for 70 ns. Theoretical scattering profiles in the q range $0 < q < 0.3$ Å^{-1} for 7,000 frames taken from the trajectory were calculated using CRYSOL (7).

RbBP5$_{NTD}$: The homology model of the WD40 domain of RBBP5 was generated automatically using the Robetta server (15). The server used RosettaCM comparative modelling protocol (16) with the yeast EIF3 subunit I (*PDBID*: 4U1E) selected as a template. The resulting model is consistent with all 7 experimental intradomain cross-links collected for the WDR5-RbBP5-MLL1$_{\text{WIN-SET}}$ trimeric complex. The homology model was used as the starting structure for all-atom MD simulations containing RbBP5$_{\text{NTD}}$. An MD trajectory of 20ns was generated and theoretical scattering profiles in the q range $0 < q < 0.3$ Å^{-1} for 2000 frames taken from the trajectory were calculated using FoXs (8). The domain keeps its structure along the trajectory within 3.5 Å of backbone r.m.s.d. to the initial model. The calculated scattering curves were averaged over the entire ensemble of structures using the optimal weights for each ensemble member obtained with the SES method, and this average profile was compared with the experimental scattering data.

RbBP5: The initial ensemble for SES analysis of RbBP5 was generated using RANCH (17) using the homology model for the WD40 domain, and assuming the N-terminal RbBP5$_{1\text{-}24}$ and C-terminal RbBP5$_{325\text{-}538}$ regions to be disordered. The ensemble consists of 20,000 models with random conformation of the flexible regions. The theoretical scattering profile for each member of the ensemble was calculated in the q range $0 < q < 0.3$ Å^{-1} using CRYSOL (7).

WDR5-MLL1 $_{\boldsymbol{WIN\text{-}SET}}$***:*** A pool of possible conformations of the dimeric complex was generated based on the crystal structure of the WDR5-WIN complex (18) (*PDBID*: 3EMH). 30,000 random configurations of the SET domain tethered to WDR5 via the WIN motif and flexible linker consisting or 46 amino acids (*i.e.* MLL1$_{3771\text{-}3817}$) was generated using RANCH (17). Theoretical scattering profiles were calculated in the q range $0 < q < 0.25$ Å^{-1} using CRYSOL (7).

WDR5-RbBP5: We used the model of full length RbBP5 described above to generate an ensemble of possible conformations of the WDR5-RbBP5 complex with RANCH (17). We assumed that RbBP5 interacts with WDR5 via the WBM motif as in the known crystal structure of sub-complex WDR5-WBM (19) (*PDBID*: 2XL2), so that the WD40 domain of RbBP5 and the WDR5-WBM sub-complex, both considered to be rigid in the simulations, are connected by a flexible linker consisting of 48 residues (i.e. RbBP5$_{325\text{-}372}$). The theoretical scattering profile for each of the 30,000 generated random conformations of WDR5-RbBP5 was calculated in the q range $0 < q < 0.20$ Å^{-1} using CRYSOL (20).

WDR5-RbBP5-MLL1 $_{\boldsymbol{WIN\text{-}SET}}$**:** The initial ensemble of possible conformations for the trimer was obtained in three steps. (**i**) First, rigid-body modeling of the complex using CORAL (21) was performed. The known ordered regions of the complex, which are assumed to be rigid in this phase of the simulations, consist of the known structures of the MLL1 SET domain (14), the WIN-WDR5-WBM subcomplex (22) (*PDBID*: 3P4F), and our homology model of the WD40 domain of RbBP5. The rest of the complex (~31% of all residues) are assumed to be flexible and are modeled by chains of dummy residues. The experimental interdomain cross-links data were taken onto account in the CORAL calculations by introducing six C_α - C_α distance restraints (see Table S2) with an upper bound of 30 Å. CORAL tries to build a single conformation of the complex that fits SAXS data under the imposed constraints. Upon performing multiple CORAL runs, we generated a number of different conformations of the complex that fit SAXS data, with $\chi_{SAXS} \sim 0.9$. Although the resulting conformations have different interdomain arrangements, the relative position of the SET and WD40 domain of RbBP5 are well defined. (**ii**) Second, we "refined" the best CORAL models by carrying out all-atom molecular dynamic simulations. The initial conformation for MD simulations was constructed from the CORAL model by building an all-atom reconstruction model using PULCHRA (23). A 20 ns MD trajectory was generated at T = 300 K. (**iii**) Finally, we used coarse-grained MD simulations to generate a pool of possible

conformations of the trimer that are consistent with known intermolecular binary interactions and cross-links. The structures from step 2 were used to derive the native contact map of quasi-rigid regions of the complex, which determines the nonbonded part of the Go-like potential. The quasi-rigid regions include residues $WDR5_{38\text{-}330}$, $RbBP5_{29\text{-}320}$, $MLL1_{3816\text{-}3969}$, $MLL1_{3761\text{-}3767}$, $MLL1_{3785\text{-}3792}$, and $RbBP5_{374\text{-}379}$ that correspond to the WD40 domains of WDR5 and RbBP5, SET domain of MLL1, WIN motif, RBS, and WBM, respectively. To improve sampling efficiency, the coarse-grained model of the complex was simulated using the Replica Exchange method. 10 replicas were used in the simulations at temperatures ranging from 200 to 360K. After an initial equilibration period, each replica was simulated for 2,000 ns, testing for exchanges every 1 ns. Data was collected only every 500 ps. The procedure was carried out eight times. Then ~180,000 structures were clustered using the K-means method. 34,000 clusters were used as an initial ensemble for fitting to SAXS data by the SES method. Theoretical scattering profiles for each conformation in the ensemble were calculated in the q range $0 < q < 0.23$ Å^{-1} using FoXS (8). The most populated models of the selected optimal ensemble were "refined" by performing all-atom MD simulations in order to obtain more realistic conformations for the flexible parts of the system since their interpretation is limited when coarse-grained modeling is used.

All-atom molecular dynamics simulations

A modified Generalized Born implicit solvent model (24) was exploited in the MD simulations in order to accelerate sampling of the conformational space for each of the systems. All simulations used an integration step of 2 fs with fixed bonds between hydrogen atoms and heavy atoms. The temperature was controlled by carrying out Langevin dynamics with the damping coefficient set to $2\,ps^{-1}$. The cut-off for non-bonded Lennard-Jones and electrostatic interactions was set to 18 Å. The ionic strength was set to 0.15M. All simulations were performed using NAMD 2.9 code (25) with the AMBER Parm99SB parameter set (26). For residues that coordinate Zn ions, a Zinc AMBER Force Field (27) was used.

Coarse-grained molecular dynamics simulations

We used a coarse-grained model of WDR5-RbBP5-$MLL1_{WIN\text{-}SET}$ in order to enhance the sampling efficiency in the conformational space of the complex. In this model, amino acid

residues in the proteins are represented as single beads located at their C_α positions and interacting via appropriate bonding, bending, torsion-angle, and non-bonding potential. A Gō-like model (28) was employed to maintain the structured, globular domains as quasi-rigid in the simulation. For flexible regions, we adopted a simple model in which adjacent amino acid beads are joined together into a polymer chain by means of virtual bond and angle interactions with a quadratic potential:

$$V_b = K_b(b - b_0)^2 \;; \qquad V_\alpha = K_\alpha(\alpha - \alpha_0)^2$$

with the constants K_b = 50 *kcal/mol* and K_α = 1.75 *kcal/mol* and the equilibrium values b_0 = 3.8 Å and α_0 = 112° for bonds and angles, respectively. The excluded volume between nonbonded beads was treated with a pure repulsive potential:

$$V_R = \varepsilon_R(\sigma_R / r_{ij})^{12}$$

where r_{ij} is the interbead distance, σ_R = 4 Å, and ε_R = 2.0 *kcal/mol.*

The interaction between quasi-rigid domains is modeled with residue-specific pair interaction potentials that combine short-range interactions with the long-range electrostatics (as described in (29, 30). The short-range interaction is given by a Lennard-Jones 12-10-6-type potential and a simple Debye-Hückel-type potential is used for the electrostatics interaction (30). In this study we used a dielectric constant of 80 and a Debye screening length of 10 Å, which corresponds to a salt concentration of ~100 mM.

To account for the experimentally observed cross-links we introduced in the force field, an additional distance restraint term given by the potential:

$$V_{XL}(t) = \sum_{k=1}^{N_{XL}} \delta_{\xi(t)}^{k}\, V_l^k \;; \qquad V_l^k = K_{XL}/(1 + e^{-\beta(l_k(t) - l_0)})$$

The sum is over all cross-links, N_{XL} is the number of cross-links, l_k is the C_α-C_α distance for residues involved in *kth* cross-link, l_0 = 39 Å is the upper bound, β = 0.5 is the slope of the sigmoidal function, K_{XL} = 10 kcal/mol is the force constant, δ_i^k is the Kronecker delta, and $\xi(t)$ is the random digital number selected from the interval [1, N_{XL}]. We chose to keep active only

the $N_{XL}/3$ randomly selected (numbers $\xi(t)$) restraints that are updated every τ_{XL}= 0.5 ns during the MD simulation.

In-house software was developed and used for performing constant temperature molecular dynamics simulations of the coarse-grained model described above. The Andersen method (31) was used to control the temperature.

NMR Spectroscopy.

All spectra were collected at 25°C on a Bruker Avance-II spectrometer operating at 800 MHz and equipped with a $^{1}H/^{13}C/^{15}N$ cryoprobe. RbBP5 samples were buffered at pH 7.7 with 20 mM TRIS, 250 mM NaCl, 2mM β-mercaptoethanol, 2 mM DTT and 1 mM PMSF. $WDR5_{WD40}$ samples were buffered with 20 mM TRIS pH 7.4, 150 mM NaCl, 2mM DTT, 1 mM TCEP, and 0.5 mM PMSF. The final samples contained 5% D_2O with protein concentrations ranging from 100 to 350 μM. Spectra were processed with NMRPipe (32) and analyzed with SPARKY (33). $WDR5_{WD40}$ backbone shifts were assigned using the ABACUS approach (34) combined with manual analysis. Assignments were made from conventional backbone triple resonance spectra collected using non-uniform sampling and processed using multidimensional decomposition (35). For $WDR5_{WD40}$ titrations, aliquots of MLL1-WIN peptide (GSARAEVHLRKS) and RbBP5-WBM peptide (EDEEVDVTSV) were titrated into buffered ^{15}N-labeled $WDR5_{WD40}$ in molar ratios ranging from 1:1 to 1:7 (protein:peptide). The weighted chemical shift perturbations (CSPs) were calculated using following formula: Δ (ppm) = $[(\delta_{NH})^2+(\delta_N/5)^2]^{1/2}$.

Gel Filtration experiments with OICR-9429.

A calibrated Superdex 200 column was equilibrated with 20 mM Tris pH 7.7, 150 mM NaCl, 10uM $ZnCl_2$, 5mM β-mercaptoethanol, 5 mM DTT and 1 mM PMSF. ~10 μM of WDR5, $MLL1_{WIN\text{-}SET}$ and RbBP5 proteins were loaded onto a Superdex 200 column. For OICR-9429 competition studies, the column was pre-equilibrated and the WDR5-RbBP5-$MLL_{WIN\text{-}SET}$ trimer was pre-incubated with 50 μM compound.

GST Pull-down experiments

Recombinant purified MLL1-GST proteins were incubated with RbBP5 fragments in an assay buffer containing 20mM Tris pH 7.7, 150 mM NaCl, 10μM $ZnCl_2$, 5mM β-mercaptoethanol, 5

mM DTT, 1 mM PMSF at 4°C for 1 hour, followed by incubation with 100 μL of glutathione-Sepharose beads (GE Healthcare) for an additional 1 hour. The concentration of the RbBP5 fragments was roughly double that of the MLL constructs. The mixtures were transferred to micro-columns and extensively washed with assay buffer. Bound proteins were eluted with 30 mM reduced glutathione and detected by SDS-PAGE and Coomassie staining.

Histone methyltransferase assay:

Activity assays were performed in 50 mM Tris-HCl, pH 8.0, 5 mM DTT and 0.01% Triton X-100, using 5 μM ^{3}H-SAM and 5 μM Biotin-H3$_{(1\text{-}25)}$. Increasing concentrations of RbBP5 were added to 200 nM of MLL1-WDR5 (with either wild-type or mutant MLL1). All reactions were incubated for 90 minutes at room temperature and the SPA method was used to determine the activities. Experiments were performed in triplicate. To test the effect of OICR-9429 on the MLL1 trimeric complex, increasing concentrations of OICR-9429 was incubated with 200 nM MLL1$_{\text{WIN-SET}}$-WDR5 for 20 min before adding 400 nM RbBP5. The activity of the complex was measured as above.

Sequence-based prediction of backbone flexibility:

We used the DynaMine web server (http://dynamine.ibsquare.be) to perform a sequence-based predictor of protein backbone dynamics. DynaMine (36) is able to accurately distinguish regions of different structural organization within proteins, such as folded domains and disordered linkers. DynaMine is based on a simple linear regression approach, and the predicted values of S2 order parameters are not transformed into a 0-1 scale, therefore due to the linear nature of the method and the fact that it was trained on soluble proteins, regions with unexpected sequence compositions can get scores above 1. The predictions are not transformed or rescaled to ensure that the resulting values make sense on the absolute scale.

Table S1. Binding constants for different interactions among components of the MLL1 trimer.

Interactors	K_D (μM)	Method[2]	Source
WDR5 – $RbBP5_{371\text{-}410}$	2.0 ± 0.3	ITC	22
WDR5 – RbBP5 (WBM peptide)	1.8 ± 0.1	ITC	19
WDR5 – MLL1 (WIN peptide)	1.7 ± 0.1	ITC	14, 35
WDR5 – $MLL1_{WIN\text{-}SET}$	0.12	AU	36
WDR5 – RbBP5	2.44	AU	36
$MLL1_{SET}$ – ($RbBP5_{AS+ABM}$ – $ASH2L_{SPRY}$)	126 ± 15	FP	37
$RbBP5_{NTD}$ – $MLL1_{RBS\text{-}SET}$	8.0	BLI	This paper
[1]$RbBP5_{NTD\text{-}AS+ABM}$ – $MLL1_{RBS\text{-}SET}$	1.2	BLI	This paper
WDR5 – RbBP5	0.3	BLI	This paper

[1]K_D obtained with very poor BLI data (not reliable)

[2]Abbreviations are: ITC, isothermal calorimetry; AU, analytical ultracentrifugation; FP, fluorescence polarization; BLI, biolayer interferometry

Table S2. Experimental interprotein cross-links[a] collected for WDR5-RbBP5-MLL1$_{WIN\text{-}SET}$ used in modelling.

Protein 1	Protein 2
**Cross-links between structured domains:**	
$MLL1_{3828}$	$RbBP5_{288}$
$MLL1_{3846}$	$RbBP5_{288}$
$MLL1_{3870}$	$RbBP5_{288}$
$MLL1_{3870}$	$RbBP5_{244}$
$MLL1_{3846}$	$WDR5_{46}$
$RbBP5_{60}$	$WDR5_{159}$
**Cross-links within flexible regions:**	
$MLL1_{3749}$	$WDR5_{7}$
$MLL1_{3749}$	$WDR5_{70}$
$MLL1_{3749}$	$WDR5_{46}$
$MLL1_{3749}$	$RbB5_{288}$
$MLL1_{3749}$	$RbB5_{505}$
$MLL1_{3749}$	$RbB5_{517}$

a) Residues in disordered regions are colored blue.

Table S3. Experimental intraprotein cross-links[a] collected for WDR5-RbBP5-MLL1$_{WIN-SET}$ which were used in the modelling.

Protein 1	Protein 2
**Cross-links within flexible regions:**	
MLL1$_{3828}$	MLL1$_{3784}$
MLL1$_{3828}$	MLL1$_{3804}$
MLL1$_{3870}$	MLL1$_{3784}$
MLL1$_{3870}$	MLL1$_{3772}$
MLL1$_{3870}$	MLL1$_{3749}$
MLL1$_{3846}$	MLL1$_{3784}$
MLL1$_{3870}$	MLL1$_{3749}$
MLL1$_{3924}$	MLL1$_{3749}$
MLL1$_{3749}$	MLL1$_{3804}$
RbBP5$_{129}$	RbBP5$_{474}$
RbBP5$_{172}$	RbBP5$_{488}$
RbBP5$_{172}$	RbBP5$_{505}$
RbBP5$_{172}$	RbBP5$_{517}$
RbBP5$_{244}$	RbBP5$_{495}$
RbBP5$_{244}$	RbBP5$_{500}$
RbBP5$_{244}$	RbBP5$_{517}$
RbBP5$_{244}$	RbBP5$_{502}$
RbBP5$_{288}$	RbBP5$_{500}$
RbBP5$_{288}$	RbBP5$_{517}$

a) Residues in disordered regions are colored blue.

References

1. Konarev,P.V., Petoukhov,M.V., Volkov,V.V. and Svergun,D.I. (2006) ATSAS 2.1, a program package for small-angle scattering data analysis. *J. Appl. Crystallogr.*, **39**, 277–286.

2. Feigin,L.A. and Svergun,D.I. (1987) Structure analysis by small-angle X-ray and neutron scattering. In. Plenum Press.

3. Rambo,R.P. and Tainer,J.A. (2013) Accurate assessment of mass, models and resolution by small-angle scattering. *Nature*, **496**, 477–481.

4. Franke,D. and Svergun,D.I. (2009) DAMMIF, a program for rapid ab-initio shape determination in small-angle scattering. *J. Appl. Crystallogr.*, **42**, 342–346.

5. Volkov,V.V. and Svergun,D.I. (2003) Uniqueness of ab initio shape determination in small-angle scattering. *J. Appl. Crystallogr.*, **36**, 860–864.

6. Kozin,M.B. and Svergun,D.I. (2001) Automated matching of high- and low-resolution structural models. *J. Appl. Crystallogr.*, **34**, 33–41.

7. Svergun,D., Barberato,C. and Koch,M.H.J. (1995) *CRYSOL* – a Program to Evaluate X-ray Solution Scattering of Biological Macromolecules from Atomic Coordinates. *J. Appl. Crystallogr.*, **28**, 768–773.

8. Schneidman-Duhovny,D., Hammel,M. and Sali,A. (2010) FoXS: a web server for rapid computation and fitting of SAXS profiles. *Nucleic Acids Res.*, **38**, W540-544.

9. Leitner,A., Walzthoeni,T. and Aebersold,R. (2014) Lysine-specific chemical cross-linking of protein complexes and identification of cross-linking sites using LC-MS/MS and the xQuest/xProphet software pipeline. *Nat. Protoc.*, **9**, 120–137.

10. Chambers,M.C., Maclean,B., Burke,R., Amodei,D., Ruderman,D.L., Neumann,S., Gatto,L., Fischer,B., Pratt,B., Egertson,J., *et al.* (2012) A cross-platform toolkit for mass spectrometry and proteomics. *Nat. Biotechnol.*, **30**, 918–920.

11. Walzthoeni,T., Claassen,M., Leitner,A., Herzog,F., Bohn,S., Förster,F., Beck,M. and Aebersold,R. (2012) False discovery rate estimation for cross-linked peptides identified by mass spectrometry. *Nat. Methods*, **9**, 901–903.

12. Combe,C.W., Fischer,L. and Rappsilber,J. (2015) xiNET: cross-link network maps with residue resolution. *Mol. Cell. Proteomics MCP*, **14**, 1137–1147.

13. Berlin,K., Castañeda,C.A., Schneidman-Duhovny,D., Sali,A., Nava-Tudela,A. and Fushman,D. (2013) Recovering a representative conformational ensemble from underdetermined macromolecular structural data. *J. Am. Chem. Soc.*, **135**, 16595–16609.

14. Southall,S.M., Wong,P.-S., Odho,Z., Roe,S.M. and Wilson,J.R. (2009) Structural basis for the requirement of additional factors for MLL1 SET domain activity and recognition of epigenetic marks. *Mol. Cell*, **33**, 181–191.

15. Kim,D.E., Chivian,D. and Baker,D. (2004) Protein structure prediction and analysis using the Robetta server. *Nucleic Acids Res.*, **32**, W526-531.

16. Song,Y., DiMaio,F., Wang,R.Y.-R., Kim,D., Miles,C., Brunette,T., Thompson,J. and Baker,D. (2013) High-resolution comparative modeling with RosettaCM. *Struct. Lond. Engl. 1993*, **21**, 1735–1742.

17. Bernadó,P., Mylonas,E., Petoukhov,M.V., Blackledge,M. and Svergun,D.I. (2007) Structural characterization of flexible proteins using small-angle X-ray scattering. *J. Am. Chem. Soc.*, **129**, 5656–5664.

18. Song,J.-J. and Kingston,R.E. (2008) WDR5 interacts with mixed lineage leukemia (MLL) protein via the histone H3-binding pocket. *J. Biol. Chem.*, **283**, 35258–35264.

19. Odho,Z., Southall,S.M. and Wilson,J.R. (2010) Characterization of a novel WDR5-binding site that recruits RbBP5 through a conserved motif to enhance methylation of histone H3 lysine 4 by mixed lineage leukemia protein-1. *J. Biol. Chem.*, **285**, 32967–32976.

20. Svergun,D., Barberato,C. and Koch,M.H.J. (1995) *CRYSOL* – a Program to Evaluate X-ray Solution Scattering of Biological Macromolecules from Atomic Coordinates. *J. Appl. Crystallogr.*, **28**, 768–773.

21. Petoukhov,M.V., Franke,D., Shkumatov,A.V., Tria,G., Kikhney,A.G., Gajda,M., Gorba,C., Mertens,H.D.T., Konarev,P.V. and Svergun,D.I. (2012) New developments in the ATSAS program package for small-angle scattering data analysis. *J. Appl. Crystallogr.*, **45**, 342–350.

22. Avdic,V., Zhang,P., Lanouette,S., Groulx,A., Tremblay,V., Brunzelle,J. and Couture,J.-F. (2011) Structural and biochemical insights into MLL1 core complex assembly. *Struct. Lond. Engl. 1993*, **19**, 101–108.

23. Rotkiewicz,P. and Skolnick,J. (2008) Fast procedure for reconstruction of full-atom protein models from reduced representations. *J. Comput. Chem.*, **29**, 1460–1465.

24. Onufriev,A., Bashford,D. and Case,D.A. (2000) Modification of the Generalized Born Model Suitable for Macromolecules. *J. Phys. Chem. B*, **104**, 3712–3720.

25. Phillips,J.C., Braun,R., Wang,W., Gumbart,J., Tajkhorshid,E., Villa,E., Chipot,C., Skeel,R.D., Kalé,L. and Schulten,K. (2005) Scalable molecular dynamics with NAMD. *J. Comput. Chem.*, **26**, 1781–1802.

26. Hornak,V., Abel,R., Okur,A., Strockbine,B., Roitberg,A. and Simmerling,C. (2006) Comparison of multiple Amber force fields and development of improved protein backbone parameters. *Proteins*, **65**, 712–725.

27. Peters,M.B., Yang,Y., Wang,B., Füsti-Molnár,L., Weaver,M.N. and Merz,K.M. (2010) Structural Survey of Zinc Containing Proteins and the Development of the Zinc AMBER Force Field (ZAFF). *J. Chem. Theory Comput.*, **6**, 2935–2947.

28. Clementi,C., Nymeyer,H. and Onuchic,J.N. (2000) Topological and energetic factors: what determines the structural details of the transition state ensemble and 'en-route' intermediates for protein folding? An investigation for small globular proteins. *J. Mol. Biol.*, **298**, 937–953.

29. Kim,Y.C. and Hummer,G. (2008) Coarse-grained models for simulations of multiprotein complexes: application to ubiquitin binding. *J. Mol. Biol.*, **375**, 1416–1433.

30. Kim,Y.C., Tang,C., Clore,G.M. and Hummer,G. (2008) Replica exchange simulations of transient encounter complexes in protein-protein association. *Proc. Natl. Acad. Sci. U. S. A.*, **105**, 12855–12860.

31. Andersen,H.C. (1980) Molecular dynamics simulations at constant pressure and/or temperature. *J. Chem. Phys.*, **72**, 2384–2393.

32. Delaglio,F., Grzesiek,S., Vuister,G.W., Zhu,G., Pfeifer,J. and Bax,A. (1995) NMRPipe: a multidimensional spectral processing system based on UNIX pipes. *J. Biomol. NMR*, **6**, 277–293.

33. Goddard,T. and Kneller,D.G. (2004) SPARKY 3.

34. Lemak,A., Steren,C.A., Arrowsmith,C.H. and Llinás,M. (2008) Sequence specific resonance assignment via Multicanonical Monte Carlo search using an ABACUS approach. *J. Biomol. NMR*, **41**, 29–41.

35. Orekhov,V.Y., Ibraghimov,I. and Billeter,M. (2003) Optimizing resolution in multidimensional NMR by three-way decomposition. *J. Biomol. NMR*, **27**, 165–173.

36. Cilia,E., Pancsa,R., Tompa,P., Lenaerts,T. and Vranken,W.F. (2013) From protein sequence to dynamics and disorder with DynaMine. *Nat. Commun.*, **4**, 2741.

37. Patel,A., Dharmarajan,V. and Cosgrove,M.S. (2008) Structure of WDR5 bound to mixed lineage leukemia protein-1 peptide. *J. Biol. Chem.*, **283**, 32158–32161.

38. Patel,A., Dharmarajan,V., Vought,V.E. and Cosgrove,M.S. (2009) On the mechanism of multiple lysine methylation by the human mixed lineage leukemia protein-1 (MLL1) core complex. *J. Biol. Chem.*, **284**, 24242–24256.

39. Li,Y., Han,J., Zhang,Y., Cao,F., Liu,Z., Li,S., Wu,J., Hu,C., Wang,Y., Shuai,J., *et al.* (2016) Structural basis for activity regulation of MLL family methyltransferases. *Nature*, **530**, 447–452.
