# Supplementary material for: The MLL1 trimeric catalytic complex is a dynamic conformational ensemble stabilized by multiple weak interactions
Source: Nucleic Acids Res. 2019 Aug 10;47(17):9433–47. doi: 10.1093/nar/gkz697 (PMC6755125; doi:10.1093/nar/gkz697)
Supplement: gkz697_Supplemental_Files [file gkz697_supplemental_files.zip › MLL_complex_Supplementary_Figures_July22.pdf]

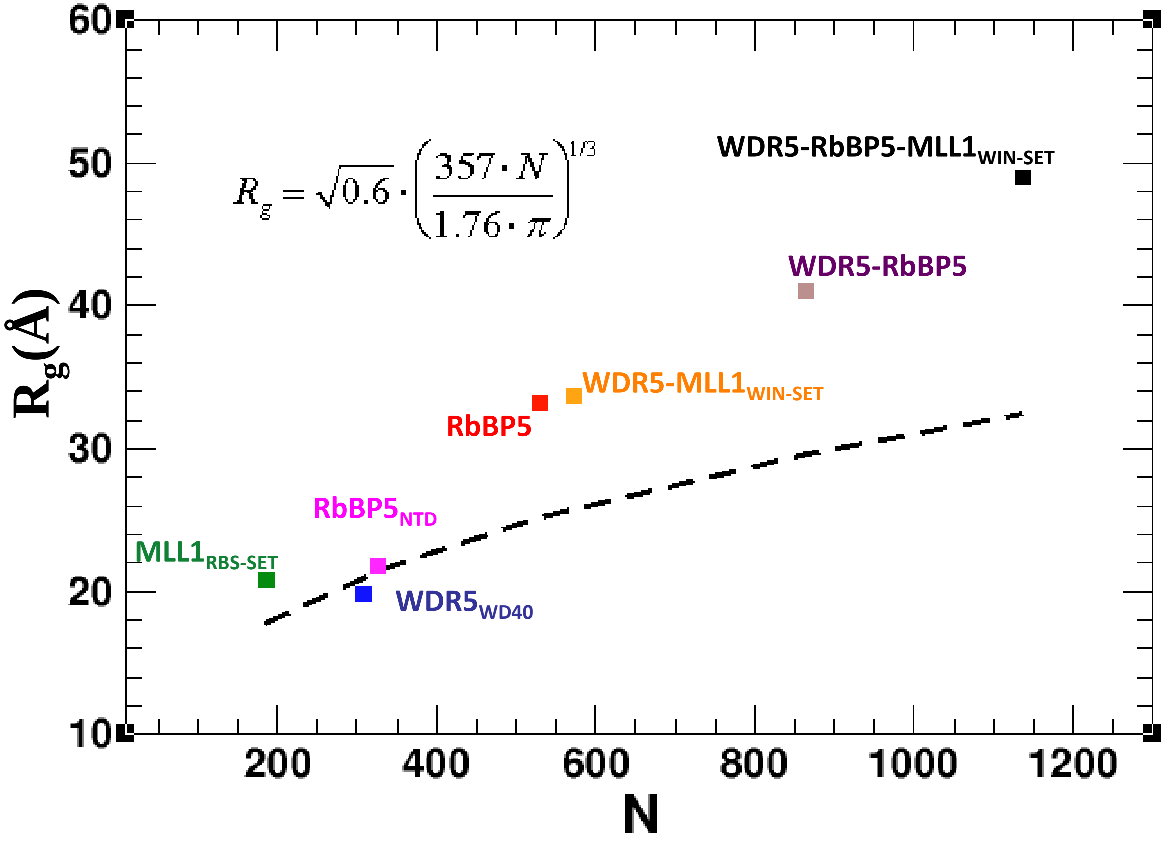


**Figure S1.**

**Comparison of the experimental Rg with the expected Rg for globular proteins.** Experimental Rg derived from SAXS data for MLL1$_{RBS-SET}$ (green), WDR5$_{WD40}$ (blue), RbBP5$_{NTD}$ (magenta), RbBP5 (red), WDR5-MLL1$_{WIN-SET}$ (orange), WDR5-RbBP5 (maroon), and WDR5-RbBP5-MLL$_{WIN-SET}$ (black) are shown *vs* the number of residues (N). Theoretical $R_g$ expected for a globular protein with the same molecular mass is shown by the dashed black line.

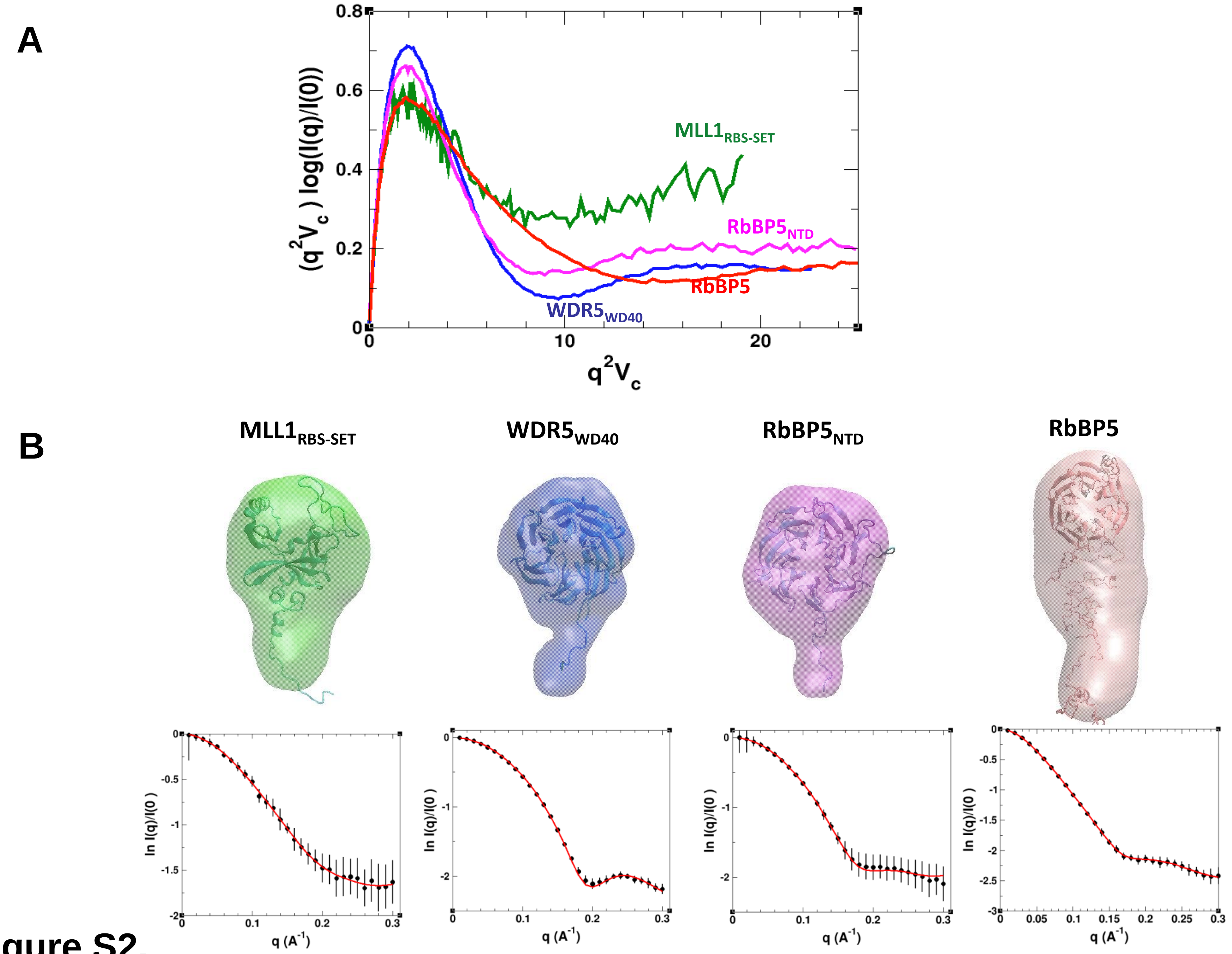

A
0.8
0.6
0.4
0.2
0
0
10
20
(q²Vc) log(I(q)/I(0))
q²Vc
MLL1RBS-SET
RbBP5NTD
RbBP5
WDR5WD40
B
MLL1RBS-SET
WDR5WD40
RbBP5NTD
RbBP5
ln I(q)/I(0)
q (A⁻¹)


**Figure S2.**

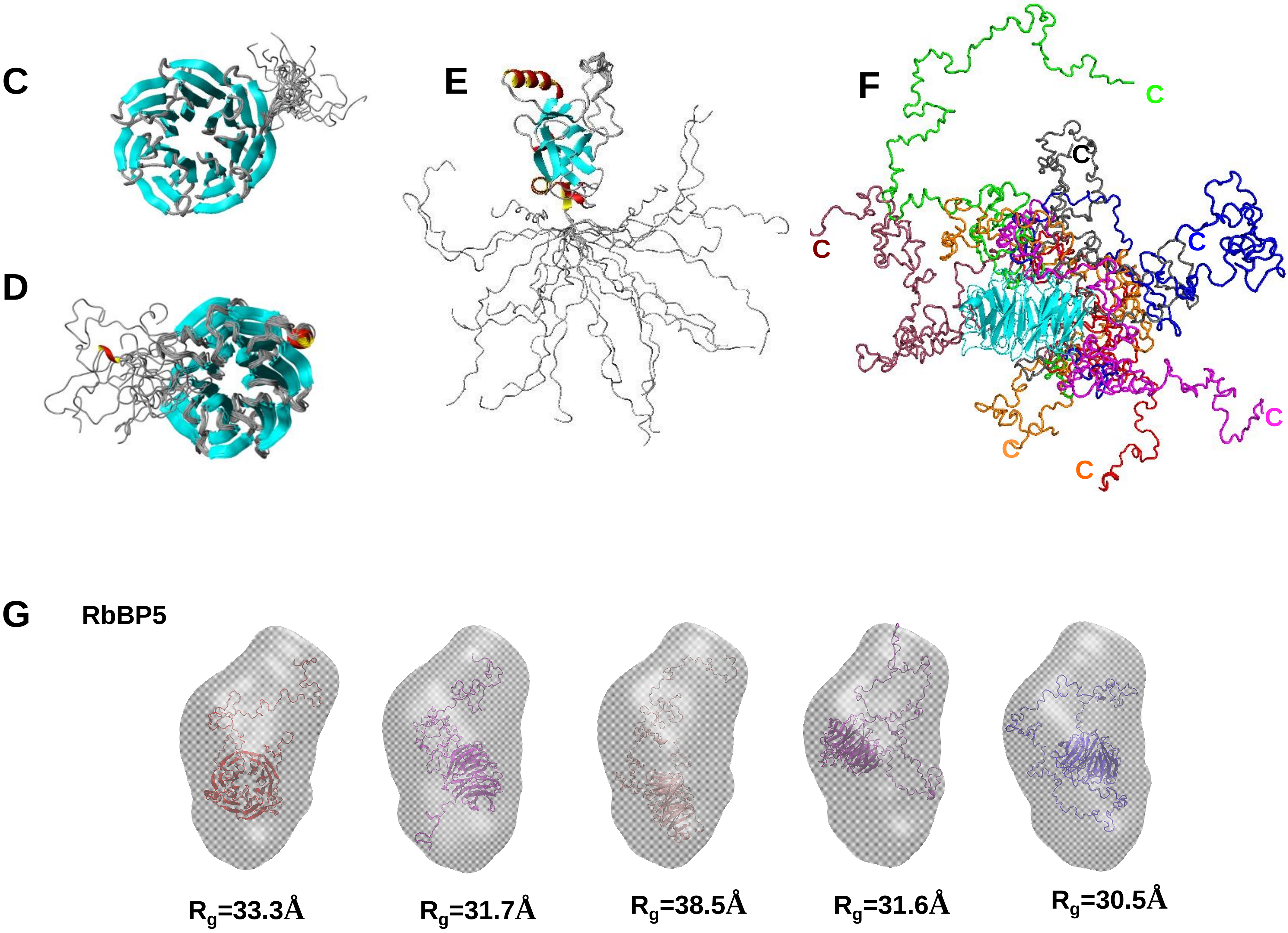


**Figure S2.**

**H**

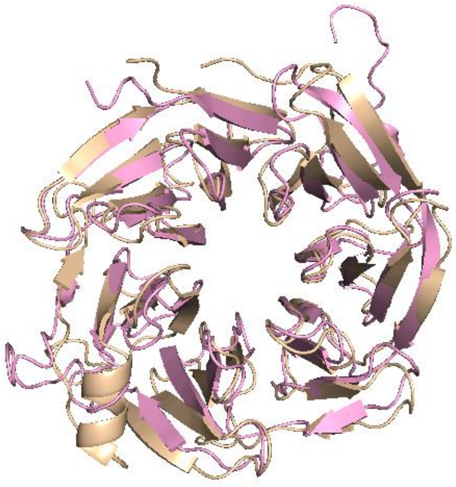

**human RbBP5$_{NTD}$ (homology model)**

**mouse RbBP5$_{NTD}$ (*PDBID*: 5OV3)**

**r.m.s.d. ~ 2.0 Å;**

**100% sequence identity**

**Figure S2. Individual components of the MLL1 core complex exhibit different degrees of flexibility. (A)** $V_c$-based Kratky plots of SAXS data for MLL1$_{RBS\text{-}SET}$ (green), WDR5$_{WD40}$ (blue), RbBP5$_{NTD}$ (magenta), and RbBP5 (red). **(B)** *Top row: Ab-initio* SAXS-predicted molecular envelopes with fitted molecular models. Filtered envelopes were calculated from fifteen runs of DAMMIF (1). Fitted models for MLL1$_{RBS\text{-}SET}$ and WDR5$_{WD40}$ are the crystal structures 2W5Y and 2H9M, respectively. For RbBP5$_{NTD}$, a homology model obtained with ROSETTA (2) is shown. For RbBP5, the five most populated models of the dynamic ensemble are shown (see also panel **G**). *Lower row:* Experimental SAXS profiles (black circles) superimposed with theoretical profiles (red) calculated for dynamic models of MLL1$_{RBS\text{-}SET}$, WDR5$_{WD40}$, RbBP5$_{NTD}$, and RbBP5 (for details see Supplementary Data). **(C-F)** An ensemble of models (with heterogeneous conformations of the flexible parts) fit the experimental profiles better than any individual protein conformation. Ribbon diagram of the optimal ensemble of: WDR5$_{WD40}$ (**C**), RbBP5$_{NTD}$ (**D**), MLL1$_{RBS\text{-}SET}$ with a flexible N-terminus **(E)**, and RbBP5 (**F**). The structured parts (cyan) of an ensemble are superimposed. Disordered parts of different models of RbBP5 are shown in different colors. (**G**) Ribbon diagrams of the five most populated conformers in the optimal ensemble of RbBP5 overlaid with the average *ab-initio* SAXS-predicted molecular envelope (grey mesh). (**H**) Comparison of the homology model of RbBP5$_{NTD}$ used in this manuscript (pink) with the crystal structure of mouse RbBP5$_{NTD}$ (orange).

A

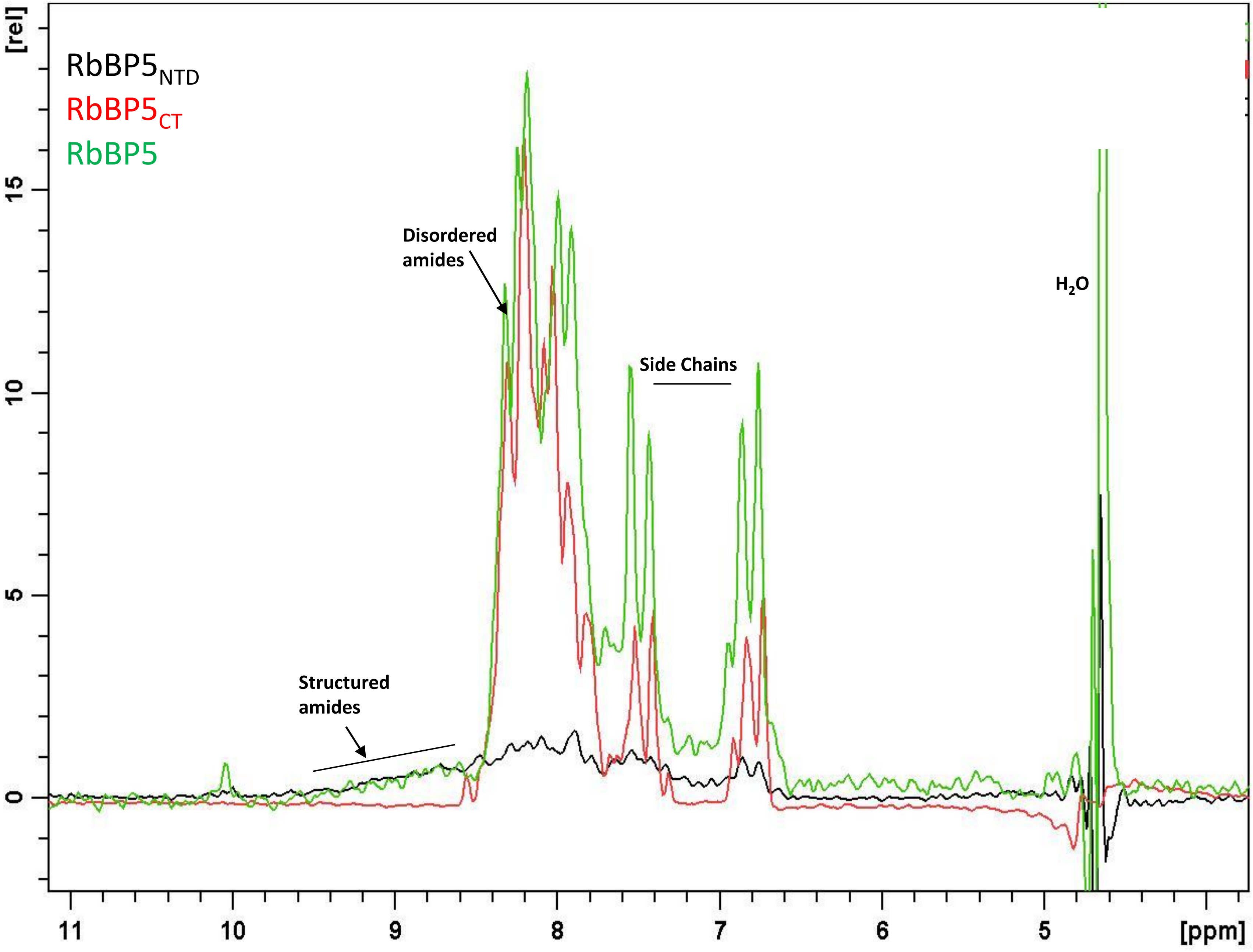
RbBP5NTD
RbBP5CT
RbBP5
Disordered amides
Side Chains
Structured amides
H2O
[rel]
15
10
5
0
11
10
9
8
7
6
5
[ppm]

Figure S3.

B

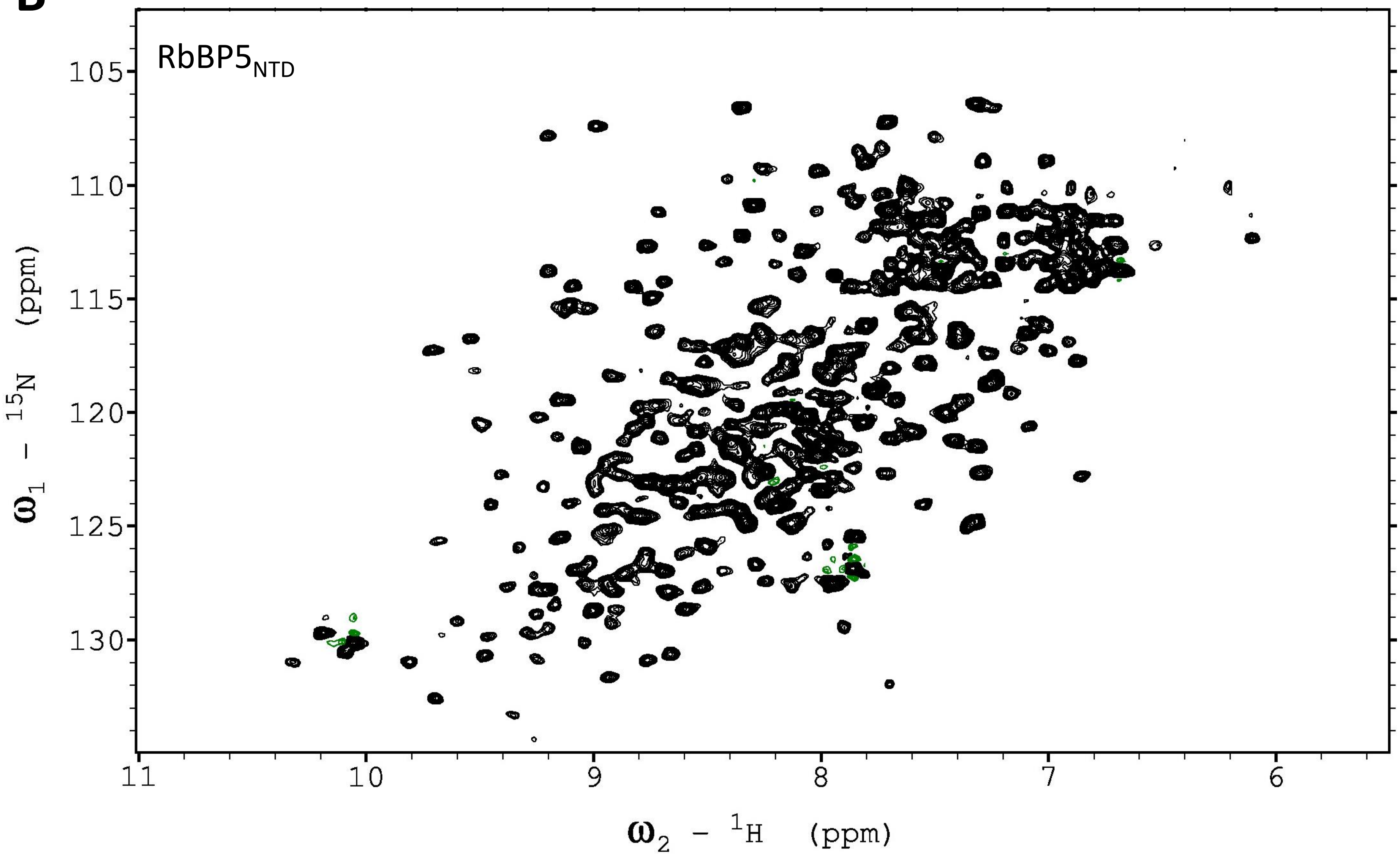

RbBP5$_{NTD}$
105
110
115
120
125
130
$\omega_1$ - $^{15}$N (ppm)
11
10
9
8
7
6
$\omega_2$ - $^{1}$H (ppm)


**Figure S3.**

C

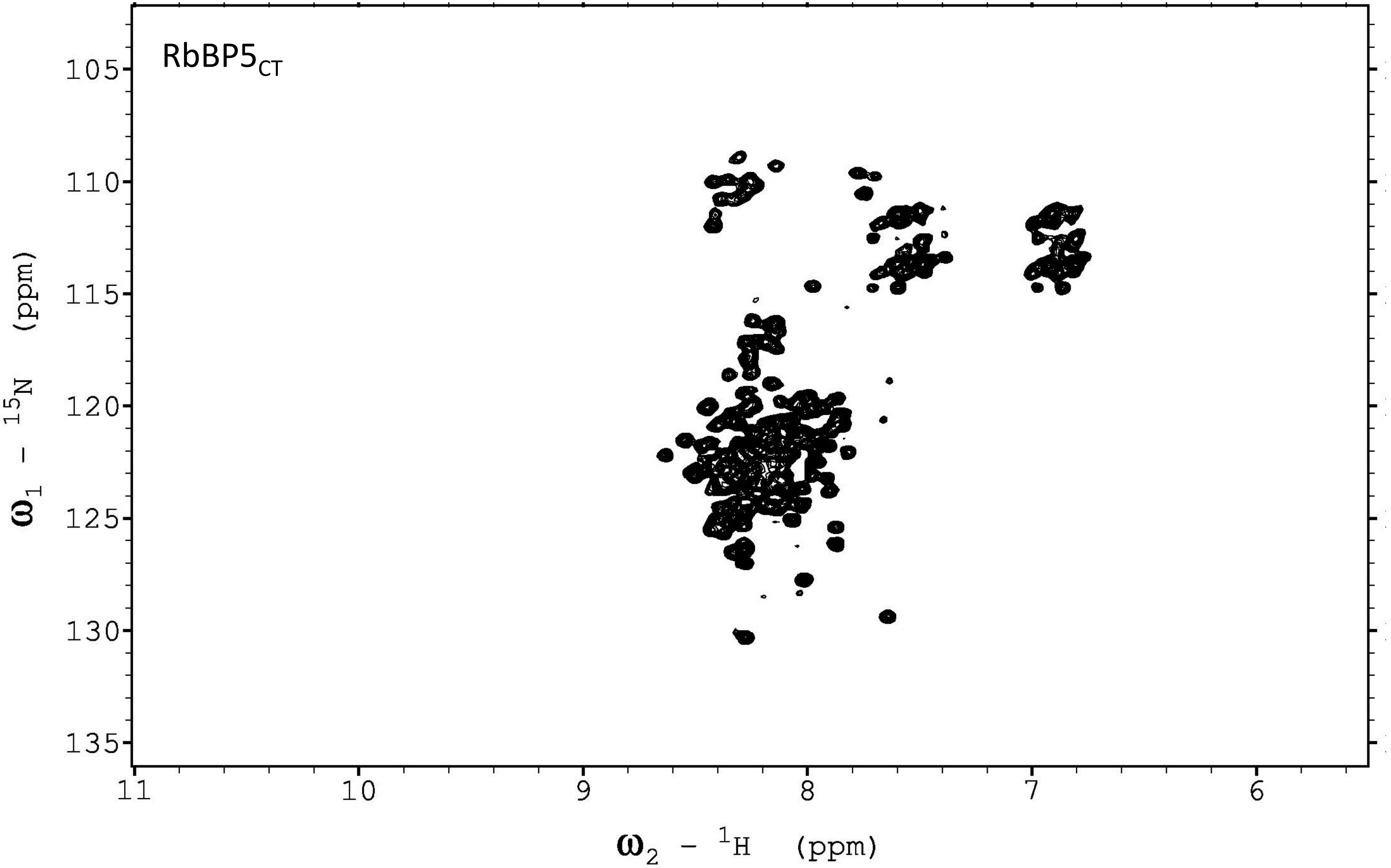
RbBP5$_{CT}$
105
110
115
120
125
130
135
$\omega_1$ - $^{15}$N (ppm)
11
10
9
8
7
6
$\omega_2$ - $^{1}$H (ppm)

**Figure S3.**

D

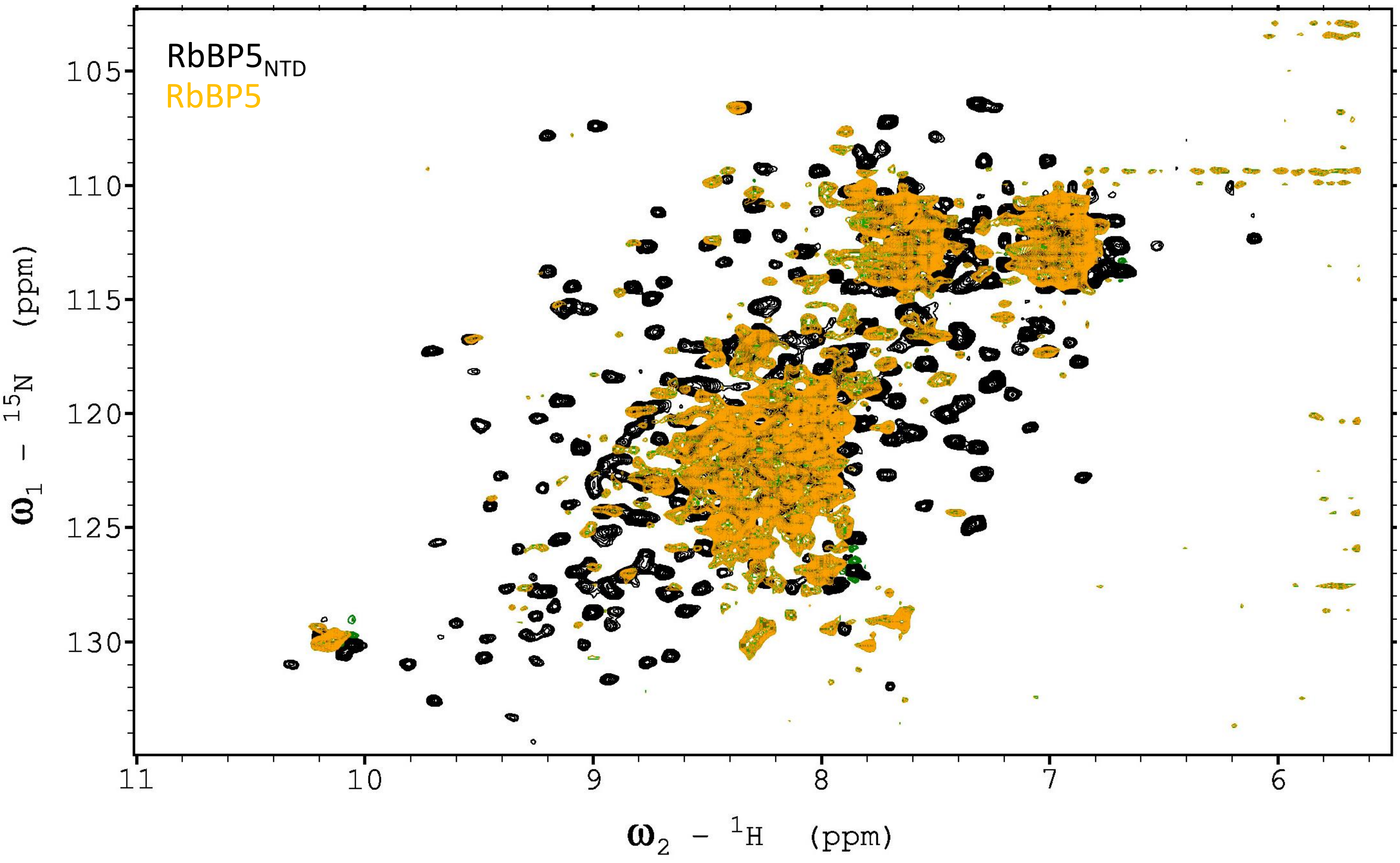

RbBP5NTD
RbBP5
105
110
115
120
125
130
ω1 - 15N (ppm)
11
10
9
8
7
6
ω2 - 1H (ppm)


Figure S3.

**E**

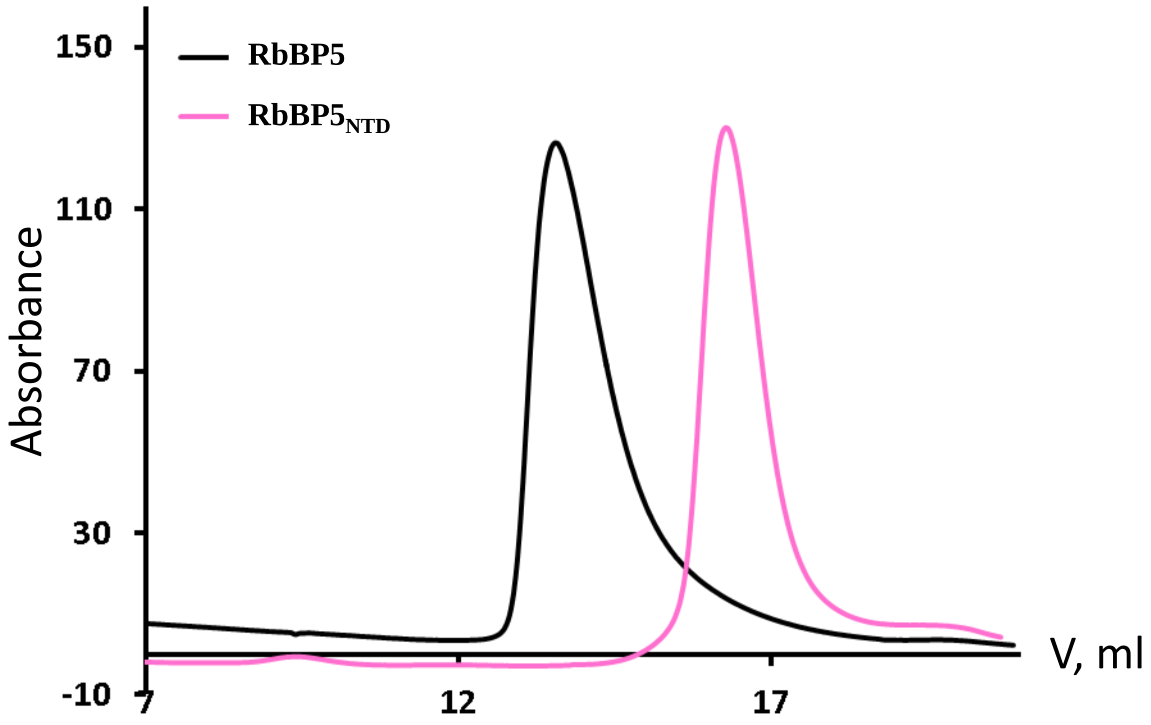


**Figure S3.**

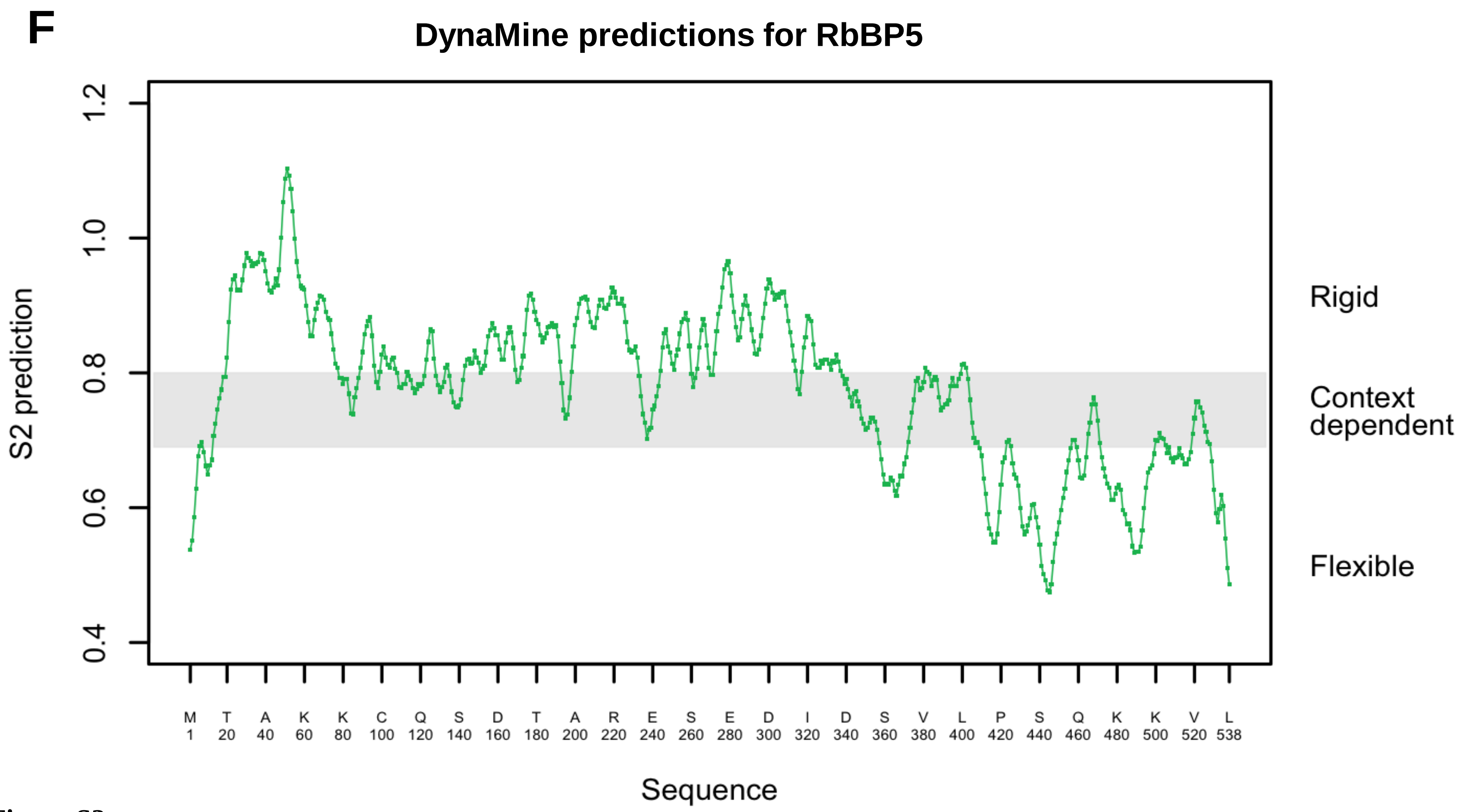


**Figure S3.**

**Structural characterization of RbBP5. (A)** Overlay of the first $^{15}$N-increment from TROSY spectra of $RbBP5_{NTD}$ (black), $RbBP5_{CT}$ (red) and RbBP5 (green). There is good overlap of RbBP5 and $RbBP5_{CT}$ traces at the centre of the spectrum (between 7.5 and 8.5 ppm) – showing that the most intense resonances in the TROSY spectrum of RbBP5 are from the unstructured CT region. The weaker/broader signals in the spectrum of RbBP5 are from the NTD and are clearly visible in the 'structured' region (8.5 to 9.5 ppm) of the traces. Complete 2D TROSY spectra for (**B**) $RbBP5_{NTD}$, (**C**) $RbBP5_{CT}$, and (**D**) overlay of $RbBP5_{NTD}$ (black) with RbBP5 (orange). **(E)** Gel filtration profiles of RbBP5 **(black)** and $RbBP5_{NTD}$ **(pink)**. RbBP5 elutes at a volume that corresponds to a higher than expected mass based on a standard curve (Ve=13.3 mL, 112.2 kDa calculated *vs*. 59.2 kDa actual) indicating a high degree of disorder. Conversely, $RbBP5_{NTD}$ elutes at a volume that corresponds to a mass that is in good agreement with its molecular weight (Ve=16.2 ml, 36.8 kDa). Note that the profiles in the panel were generated by importing raw data into Microsoft Excel and replotting. **(F)** S2 order parameters for RbBP5, predicted from the protein sequence using DynaMine (3) (freely accessed at: *http://dynamine.ibsquare.be*). Each residue is predicted to have one of three different levels of structural organization: rigid (S2 >0.8), flexible (S2<0.7), and context dependent.

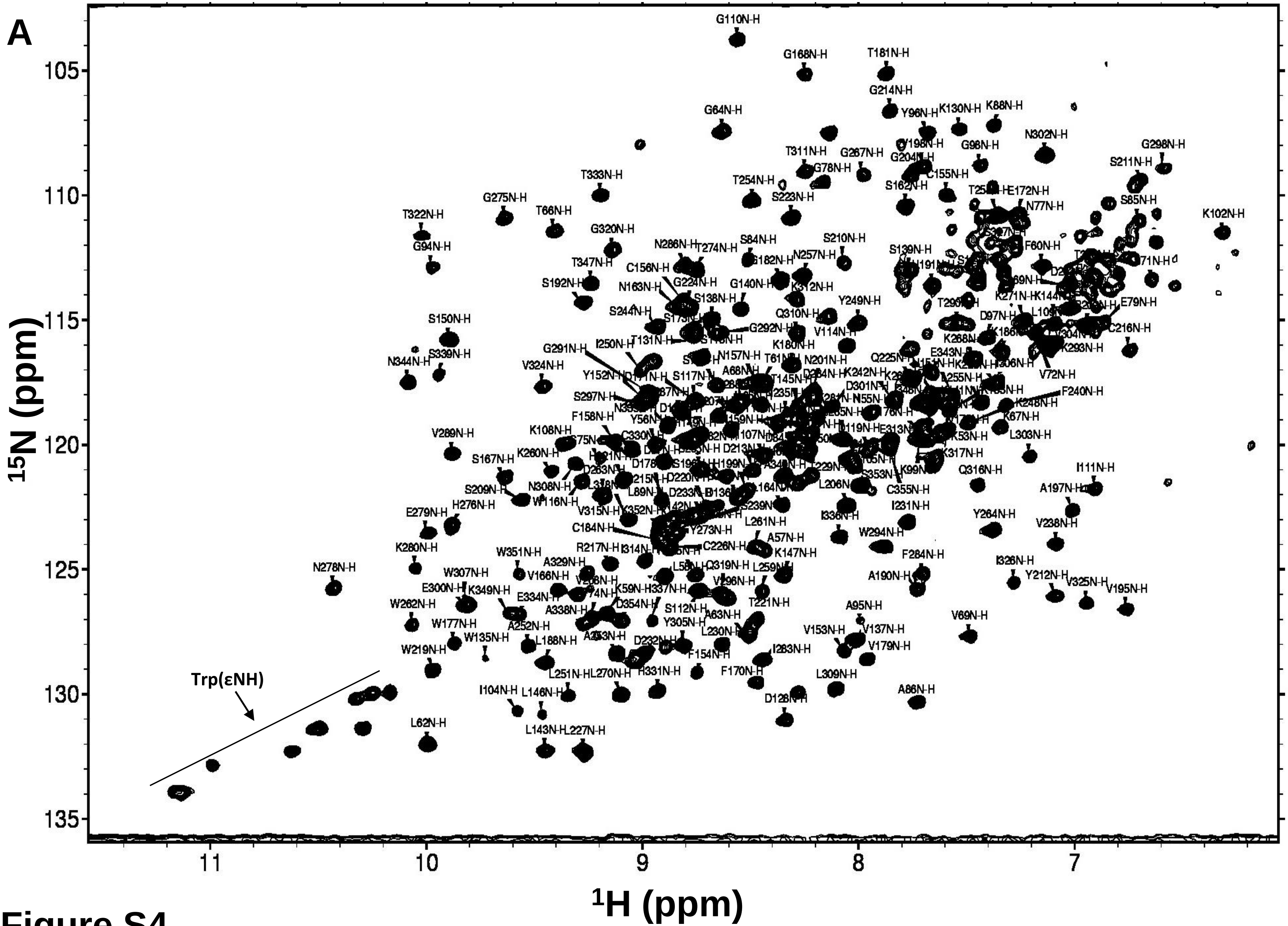

A
$^{15}$N (ppm)
$^{1}$H (ppm)
105
110
115
120
125
130
135
11
10
9
8
7
Trp(εNH)
G110N-H
G168N-H
T181N-H
G214N-H
G64N-H
Y96N-H
K130N-H
K88N-H
N302N-H
G298N-H
T311N-H
G267N-H
G98N-H
G78N-H
C155N-H
S211N-H
T333N-H
T254N-H
S162N-H
E172N-H
G275N-H
S223N-H
S85N-H
N77N-H
T66N-H
T322N-H
K102N-H
G320N-H
G94N-H
N286N-H
T274N-H
S84N-H
S210N-H
S139N-H
F60N-H
T347N-H
G182N-H
N257N-H
C156N-H
S192N-H
G224N-H
N163N-H
G140N-H
K312N-H
S138N-H
Y249N-H
E79N-H
S244N-H
Q310N-H
G292N-H
D97N-H
S150N-H
V114N-H
C216N-H
T131N-H
K180N-H
G291N-H
I250N-H
K293N-H
N344N-H
S339N-H
N157N-H
N201N-H
Q225N-H
V324N-H
K242N-H
V72N-H
Y152N-H
S117N-H
D301N-H
F240N-H
S297N-H
N55N-H
K248N-H
F158N-H
K67N-H
K108N-H
K53N-H
L303N-H
V289N-H
D119N-H
E313N-H
K317N-H
S167N-H
K260N-H
D213N-H
Q316N-H
S353N-H
I111N-H
C355N-H
S209N-H
N308N-H
L206N-H
A197N-H
W116N-H
H276N-H
S239N-H
I231N-H
E279N-H
V315N-H
K352N-H
L261N-H
I336N-H
Y264N-H
C184N-H
Y273N-H
W294N-H
V238N-H
K280N-H
W351N-H
R217N-H
C226N-H
A57N-H
K147N-H
F284N-H
I326N-H
N278N-H
A329N-H
Q319N-H
L259N-H
A190N-H
Y212N-H
W307N-H
V166N-H
V296N-H
V325N-H
E300N-H
K349N-H
V195N-H
E334N-H
D354N-H
W262N-H
A338N-H
S112N-H
T221N-H
A95N-H
V69N-H
W177N-H
Y305N-H
A63N-H
A252N-H
L230N-H
V153N-H
V137N-H
W135N-H
L188N-H
W219N-H
D232N-H
F154N-H
I283N-H
V179N-H
L251N-H
L270N-H
H331N-H
F170N-H
L309N-H
I104N-H
L146N-H
D126N-H
A86N-H
L62N-H
L143N-H
L227N-H


Figure S4.

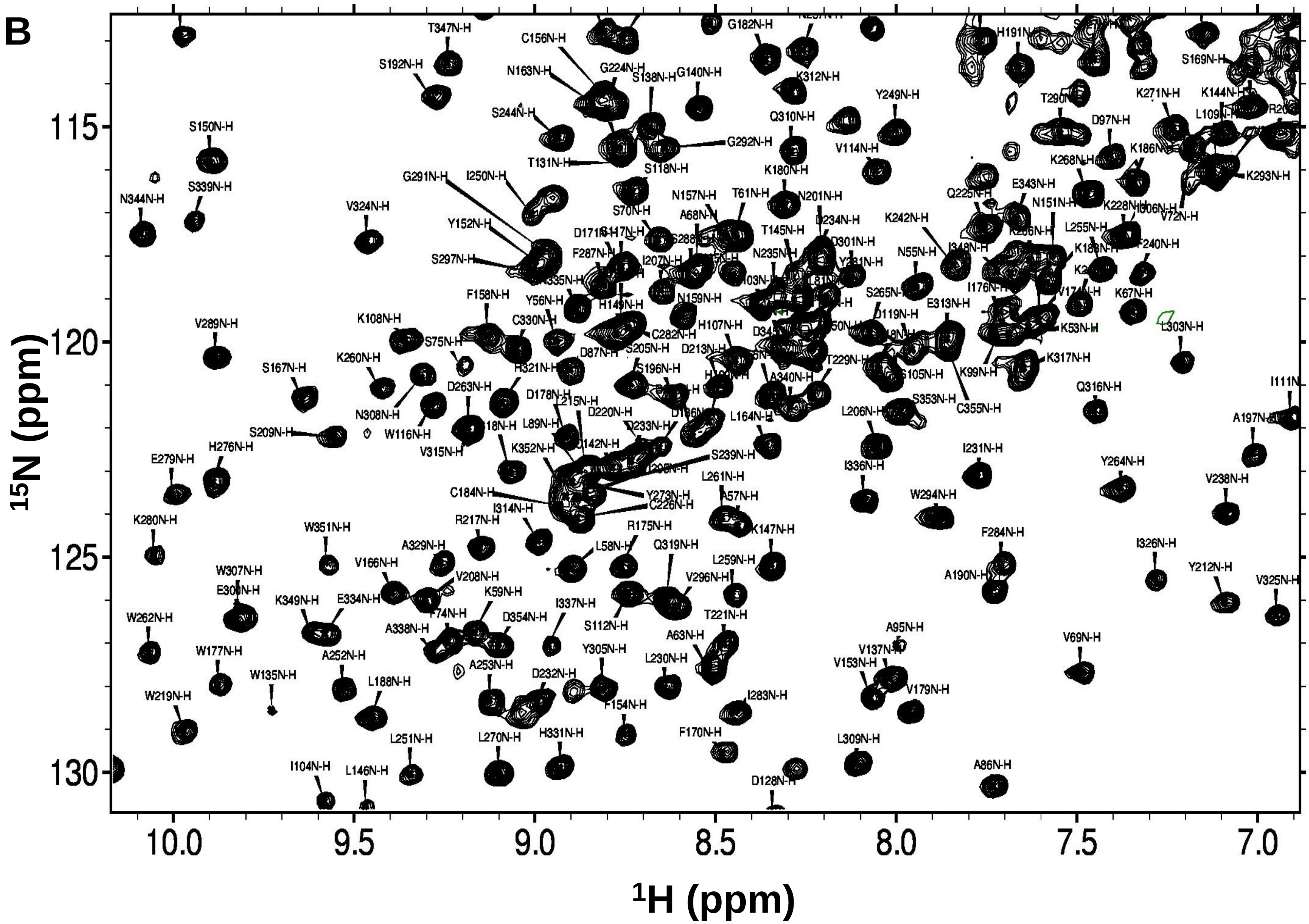
B
T347N-H
C156N-H
G182N-H
H191N-H
S192N-H
N163N-H
G224N-H
S138N-H
G140N-H
K312N-H
S169N-H
Y249N-H
K271N-H
K144N-H
S244N-H
Q310N-H
D97N-H
L109N-H
S150N-H
G292N-H
T131N-H
V114N-H
K186N-H
S118N-H
K180N-H
K268N-H
K293N-H
G291N-H
I250N-H
N344N-H
S339N-H
N157N-H
T61N-H
N201N-H
Q225N-H
E343N-H
N151N-H
K228N-H
V72N-H
V324N-H
S70N-H
A68N-N
D234N-H
K242N-H
Y152N-H
T145N-H
D301N-H
L255N-H
F240N-H
S297N-H
F287N-H
I207N-H
N235N-H
N55N-H
I348N-H
F158N-H
Y56N-H
N159N-H
S265N-H
I176N-H
K67N-H
V289N-H
K108N-H
C330N-H
H107N-H
D119N-H
E313N-H
K53N-H
L303N-H
S75N-H
C282N-H
D213N-H
K317N-H
K260N-H
D87N-H
S205N-H
T229N-H
S167N-H
H321N-H
S196N-H
A340N-H
S105N-H
K99N-H
Q316N-H
I111N
D263N-H
D178N-H
S353N-H
C355N-H
N308N-H
L215N-H
L206N-H
A197N-H
S209N-H
W116N-H
D220N-H
L164N-H
L89N-H
D233N-H
V315N-H
K352N-H
S239N-H
I231N-H
E279N-H
H276N-H
L261N-H
I336N-H
Y264N-H
V238N-H
C184N-H
Y273N-H
A57N-H
C226N-H
W294N-H
I314N-H
K280N-H
R217N-H
R175N-H
W351N-H
K147N-H
F284N-H
A329N-H
L58N-H
Q319N-H
I326N-H
V166N-H
L259N-H
W307N-H
V208N-H
V296N-H
A190N-H
Y212N-H
V325N-H
K59N-H
K349N-H
E334N-H
I337N-H
W262N-H
F74N-H
D354N-H
T221N-H
S112N-H
A95N-H
A338N-H
A63N-H
V69N-H
W177N-H
A252N-H
Y305N-H
V137N-H
L230N-H
W135N-H
A253N-H
V153N-H
D232N-H
L188N-H
V179N-H
W219N-H
I283N-H
F154N-H
F170N-H
L251N-H
L270N-H
H331N-H
L309N-H
A86N-H
I104N-H
L146N-H
D128N-H
115
120
125
130
10.0
9.5
9.0
8.5
8.0
7.5
7.0
15N (ppm)
1H (ppm)

Figure S4.

**Figure S4.**

**($^{1}H$-$^{15}N$)-TROSY spectrum of $WDR5_{WD40}$.**

Full (**A**) and central portion (*zoomed in*) (**B**) of a TROSY spectrum. Peaks are labeled with resonance assignments – 254 backbone spin systems were assigned, and these were deposited to the BMRB database (*BMRB_ID*: 27528)

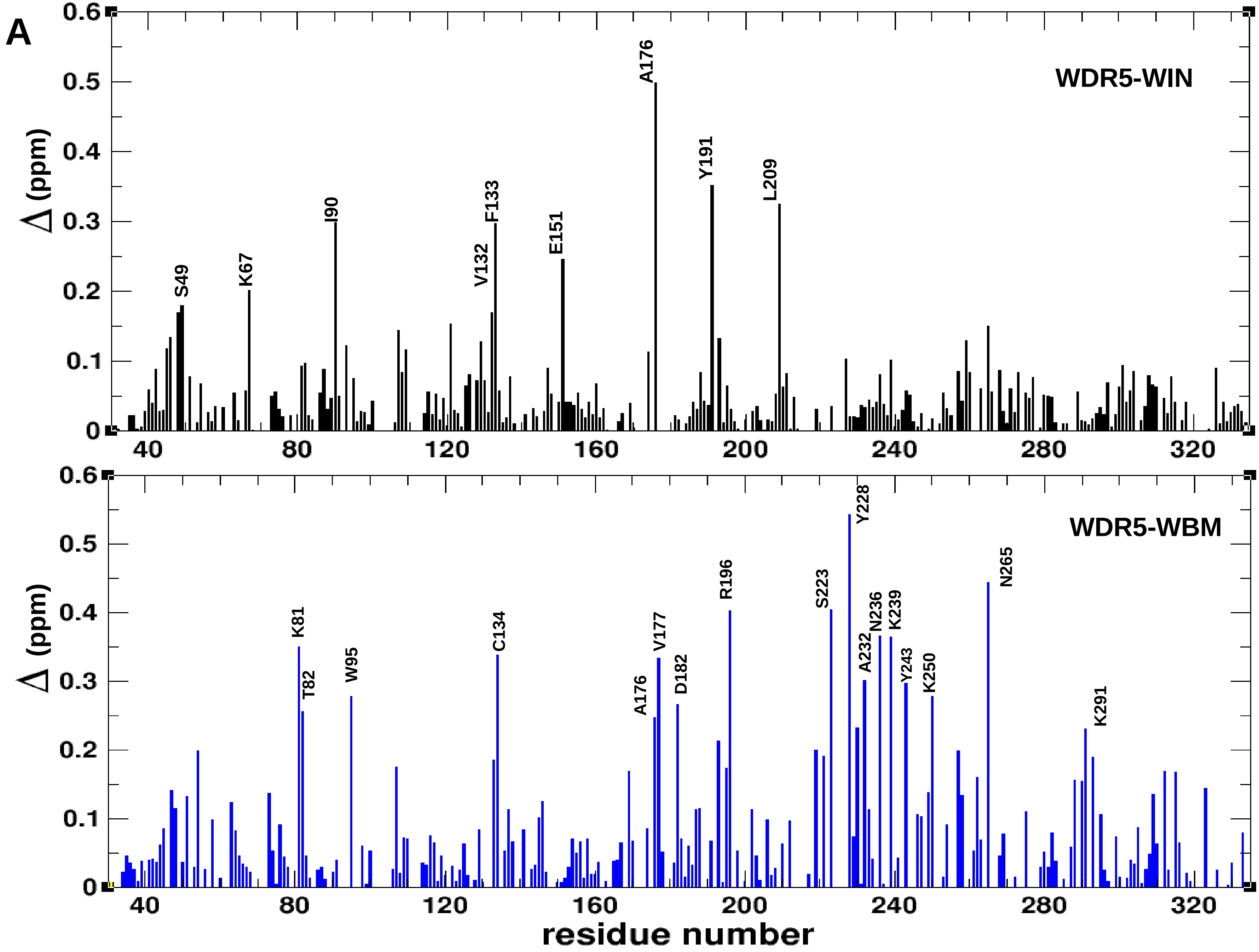
A
WDR5-WIN
Δ (ppm)
S49
K67
I90
V132
F133
E151
A176
Y191
L209
WDR5-WBM
K81
T82
W95
C134
A176
V177
D182
R196
S223
Y228
A232
N236
K239
Y243
K250
N265
K291
residue number
0
0.1
0.2
0.3
0.4
0.5
0.6
40
80
120
160
200
240
280
320

**Figure S5.**

B

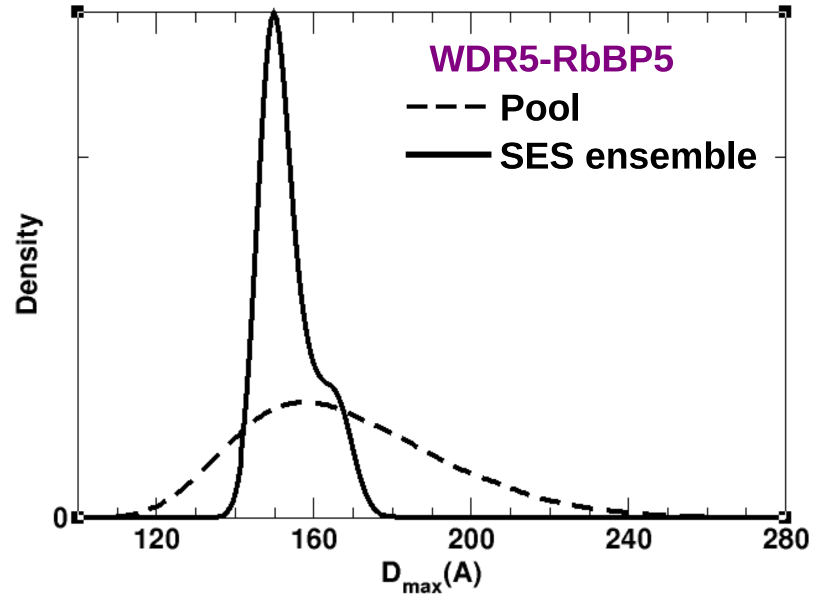
WDR5-RbBP5
Pool
SES ensemble
Density
0
120
160
200
240
280
Dmax(A)

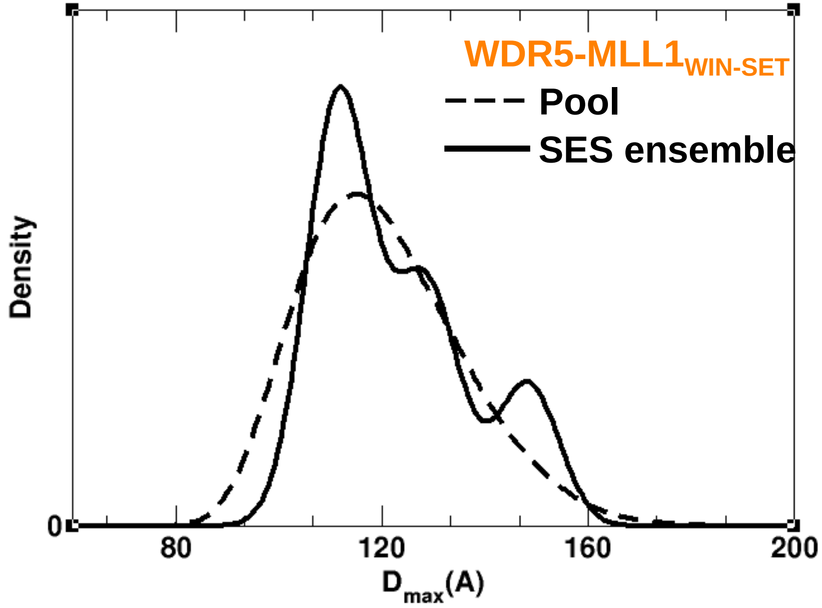
WDR5-MLL1WIN-SET
Pool
SES ensemble
Density
0
80
120
160
200
Dmax(A)

C

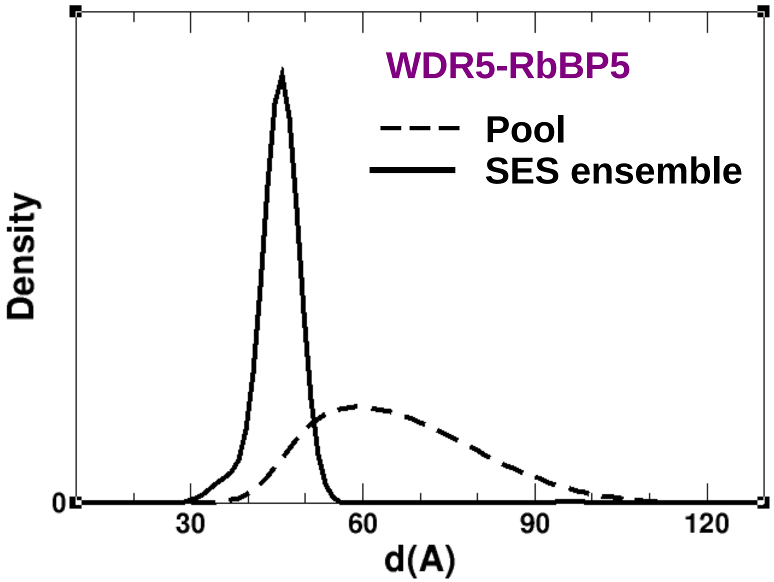
WDR5-RbBP5
Pool
SES ensemble
Density
0
30
60
90
120
d(A)

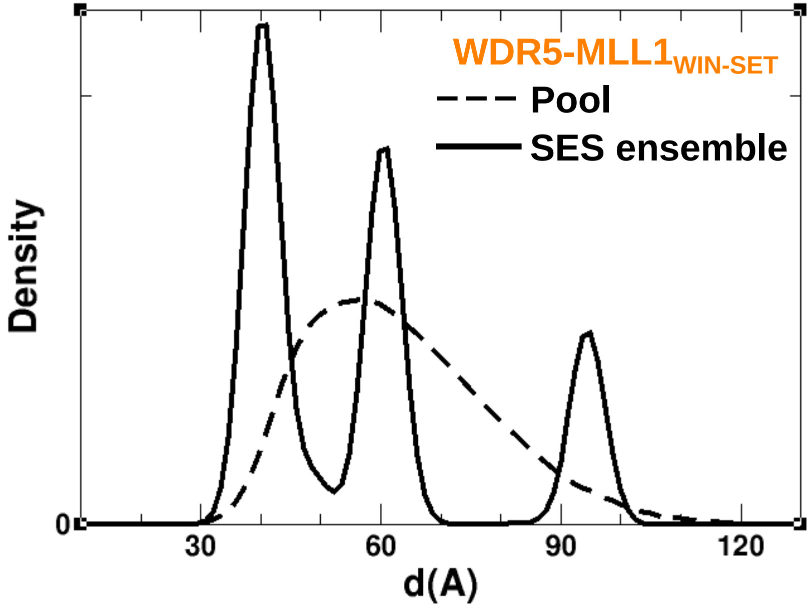
WDR5-MLL1WIN-SET
Pool
SES ensemble
Density
0
30
60
90
120
d(A)

**Figure S5.**

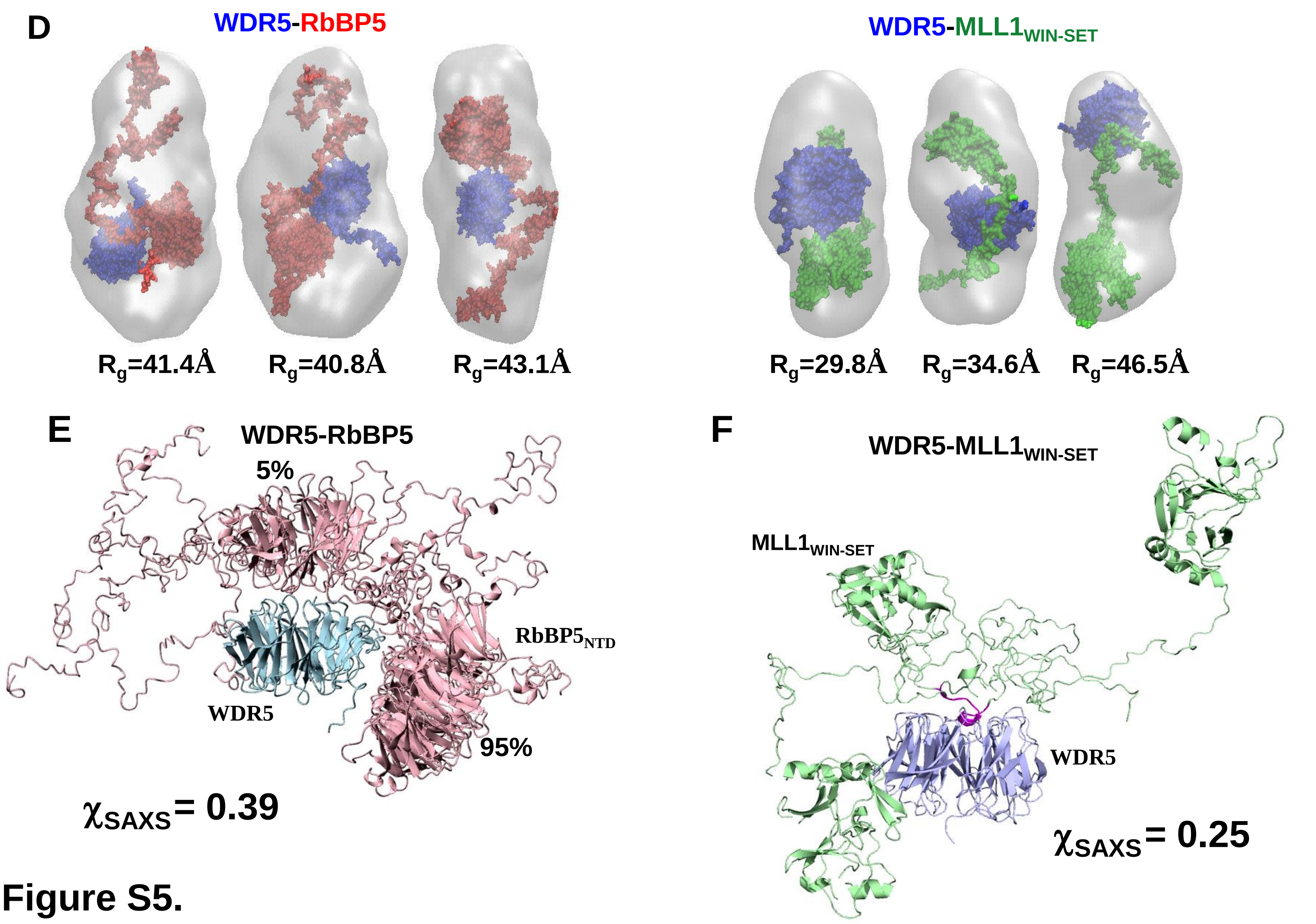

D
WDR5-RbBP5
WDR5-MLL1$_{WIN-SET}$
$R_g$=41.4Å
$R_g$=40.8Å
$R_g$=43.1Å
$R_g$=29.8Å
$R_g$=34.6Å
$R_g$=46.5Å
E
WDR5-RbBP5
5%
RbBP5$_{NTD}$
WDR5
95%
$\chi_{SAXS}$= 0.39
F
WDR5-MLL1$_{WIN-SET}$
MLL1$_{WIN-SET}$
WDR5
$\chi_{SAXS}$= 0.25


Figure S5.

G

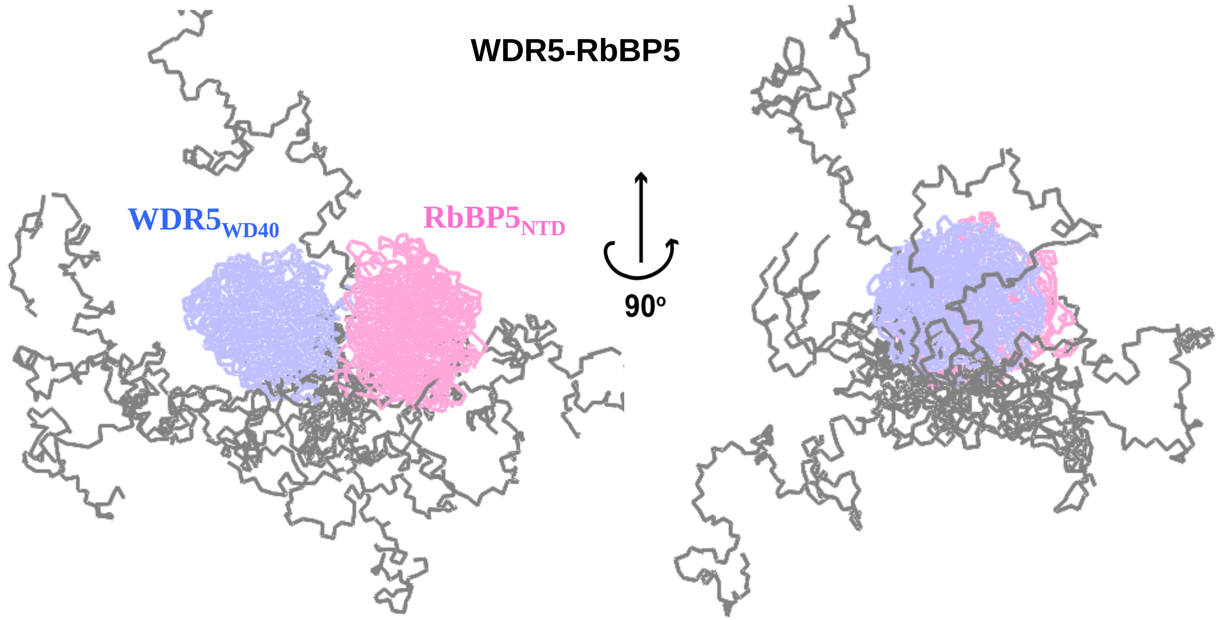
WDR5-RbBP5
WDR5WD40
RbBP5NTD
90°

H

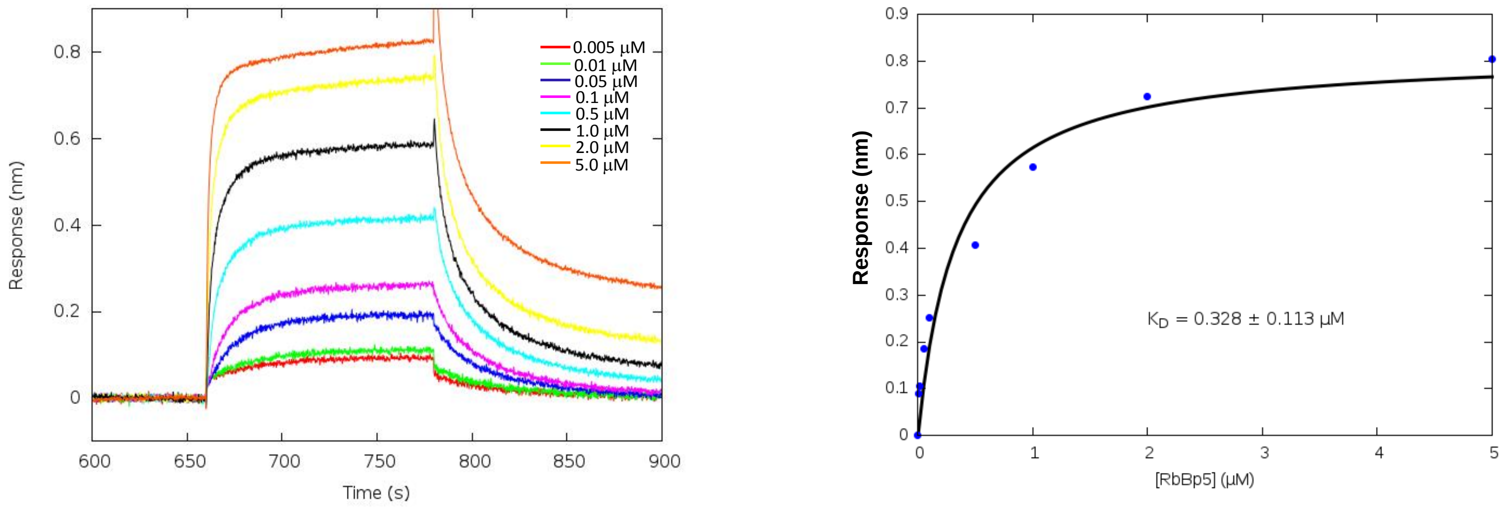
WDR5 + RbBP5
Response (nm)
Time (s)
0.005 µM
0.01 µM
0.05 µM
0.1 µM
0.5 µM
1.0 µM
2.0 µM
5.0 µM
0
0.2
0.4
0.6
0.8
600
650
700
750
800
850
900
Response (nm)
[RbBp5] (µM)
KD = 0.328 ± 0.113 µM
0
0.1
0.2
0.3
0.4
0.5
0.6
0.7
0.8
0.9
0
1
2
3
4
5

# Figure S5.

**Structural characterization of WDR5-MLL1$_{WIN\text{-}SET}$ and WDR5-RbBP5 binary complexes.** (**A**) Peak movement in ($^{1}H$-$^{15}N$)-TROSY spectra of assigned WDR5 amide resonances, resulting from WIN (top) or WBM (bottom) peptide binding. The marked residues are those with CSPs greater than 2 standard deviation units above the average CSP for all assigned resonances. (**B**) $D_{max}$ distribution for WDR5-RbBP5 (left) and WDR5-MLL1$_{WIN\text{-}SET}$ (right**)**. The distribution for the initial pool of random structures (dashed lines) and for the selected SES ensemble (solid lines) are shown. WDR5-RbBP5 favors structures that are more condensed than fully extended, while WDR5-MLL1$_{WIN\text{-}SET}$ has a higher degree of flexibility and exhibits a population of fully extended structures. (**C**) Distribution of the distance between centers of mass of WDR5 and the RbBP5 β-propeller domain (left), and WDR5$_{WD40}$ and MLL1$_{SET}$ (right) domains in the optimal ensembles of binary complexes. **(D)** Surface representation of models for the binary complex overlaid with *ab-initio* SAXS-predicted molecular envelopes (grey mesh) for WDR5-RbBP5 (left) and WDR5-MLL1$_{WIN\text{-}SET}$ (right). The three most populated models of the optimal ensemble are shown. WDR5, MLL1, and RbBP5 are colored in blue, green and red, respectively. (**E-F**) Ribbon diagram of the representative models of the optimal ensemble of WDR5-RbBP5 (**E**) and WDR5-MLL1$_{WIN\text{-}SET}$ (**F**). WDR5 structured domains of different members of an ensemble are superimposed. WDR5, RbBP5 and MLL1 are colored in blue, pink, and green, respectively. The WIN motif of MLL1 is shown in magenta. The goodness-of-fit of the optimal ensemble to SAXS data is shown by the $\chi_{SAXS}$. (**G**) Ribbon diagram of the most populated models of the optimal ensemble of WDR5-RbBP5. Both WDR5$_{WD40}$ and RBBP5$_{NTD}$ structured domains of different members of the ensemble are superimposed and shown in blue and pink, respectively. (**H**) BLI sensorgrams (left) for the binding of RbBP5 to sensor-immobilized GST-tagged WDR5, and the corresponding steady-state binding curve (right).

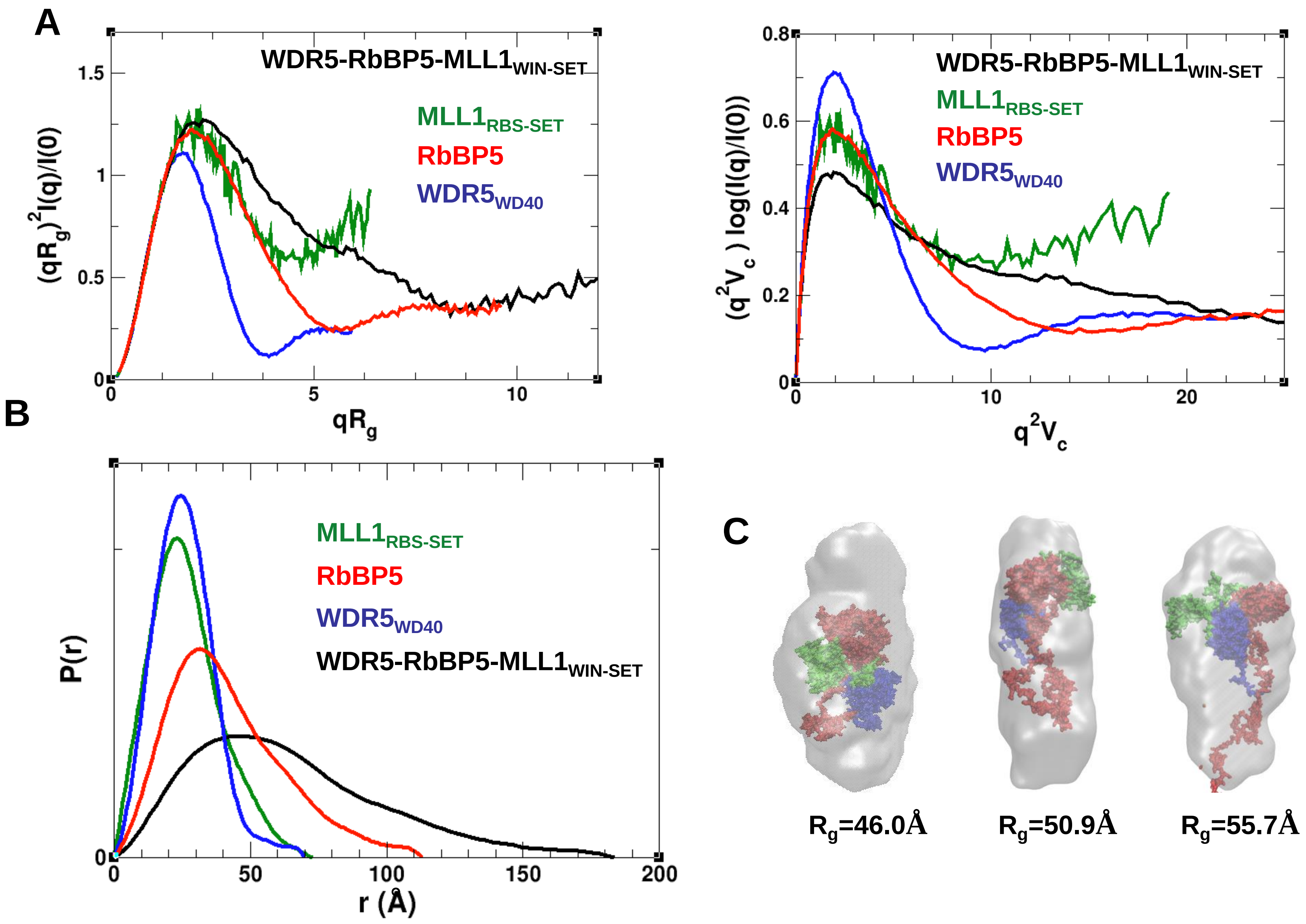
A
WDR5-RbBP5-MLL1$_{WIN-SET}$
MLL1$_{RBS-SET}$
RbBP5
WDR5$_{WD40}$
$(qR_g)^2 I(q)/I(0)$
$qR_g$
0
0.5
1
1.5
5
10
$(q^2V_c)\log(I(q)/I(0))$
$q^2V_c$
0.2
0.4
0.6
0.8
20
B
P(r)
r (Å)
50
100
150
200
C
$R_g$=46.0Å
$R_g$=50.9Å
$R_g$=55.7Å

Figure S6.

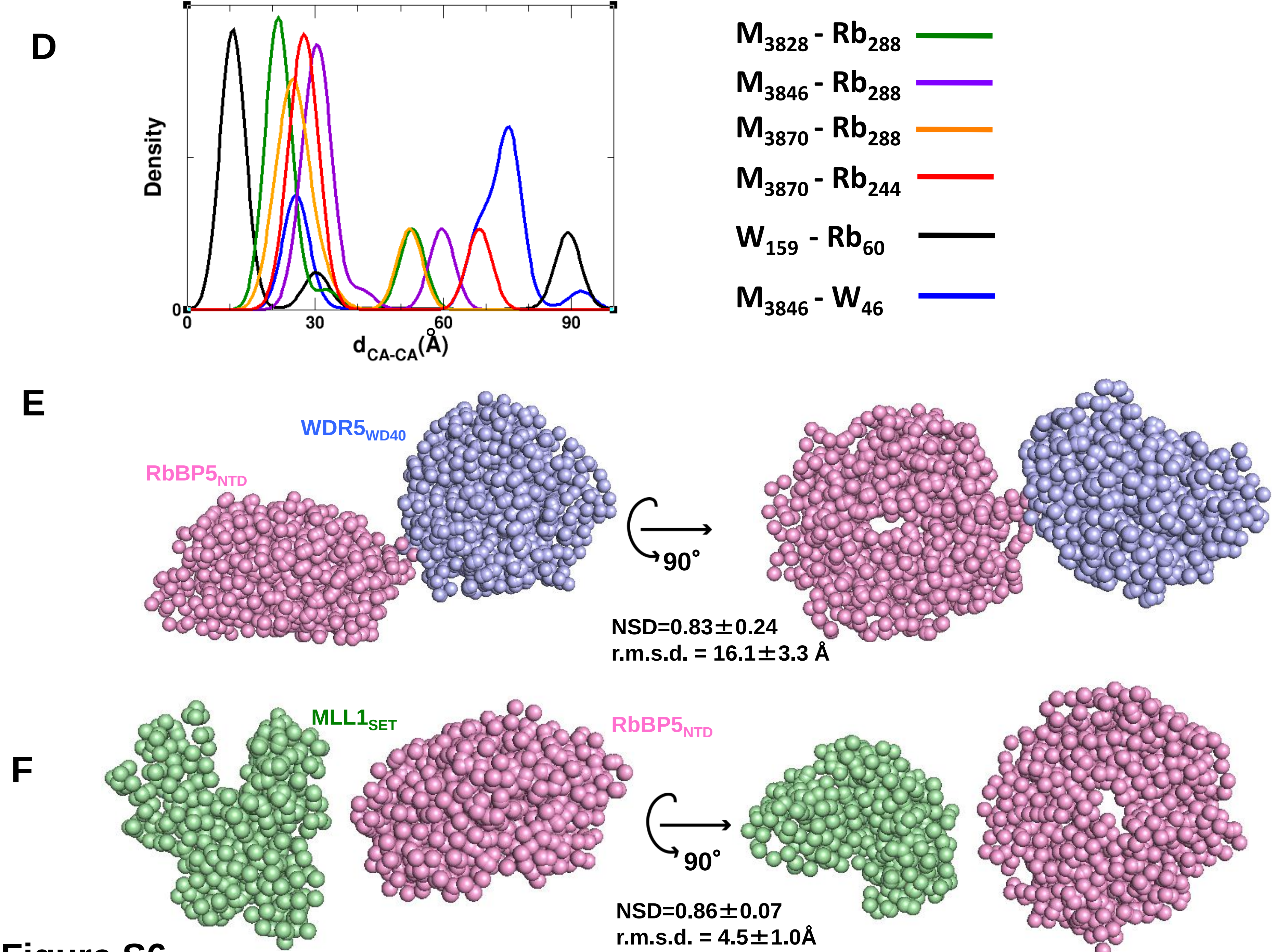
D
Density
0
0
30
60
90
$d_{CA-CA}$(Å)
$M_{3828}$ - $Rb_{288}$
$M_{3846}$ - $Rb_{288}$
$M_{3870}$ - $Rb_{288}$
$M_{3870}$ - $Rb_{244}$
$W_{159}$ - $Rb_{60}$
$M_{3846}$ - $W_{46}$
E
$WDR5_{WD40}$
$RbBP5_{NTD}$
90°
NSD=0.83±0.24
r.m.s.d. = 16.1±3.3 Å
F
$MLL1_{SET}$
$RbBP5_{NTD}$
90°
NSD=0.86±0.07
r.m.s.d. = 4.5±1.0Å

**Figure S6.**

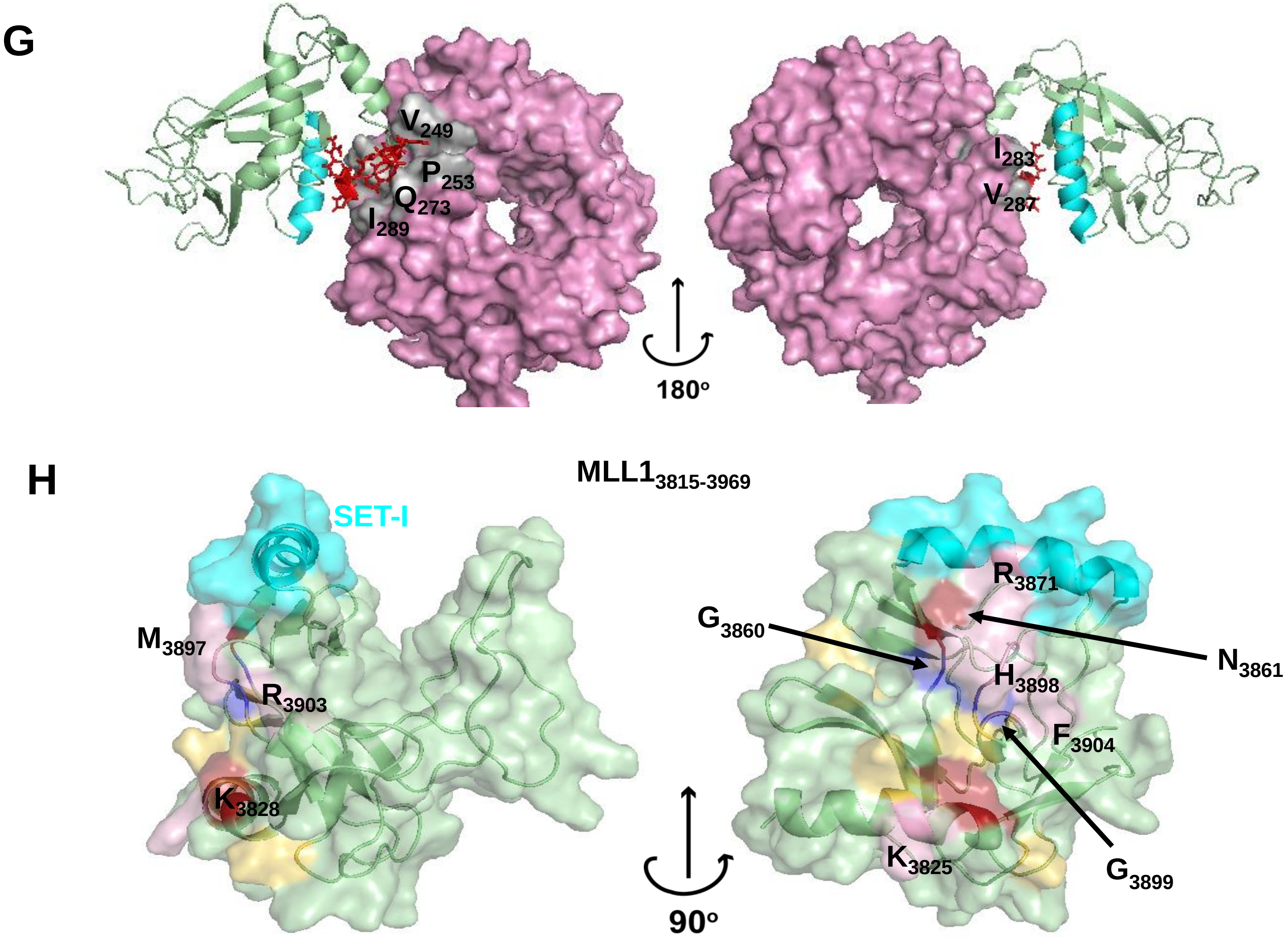
G
V249
P253
Q273
I289
I283
V287
180°
H
MLL1 3815-3969
SET-I
M3897
R3903
K3828
R3871
G3860
N3861
H3898
F3904
K3825
G3899
90°

Figure S6.

I

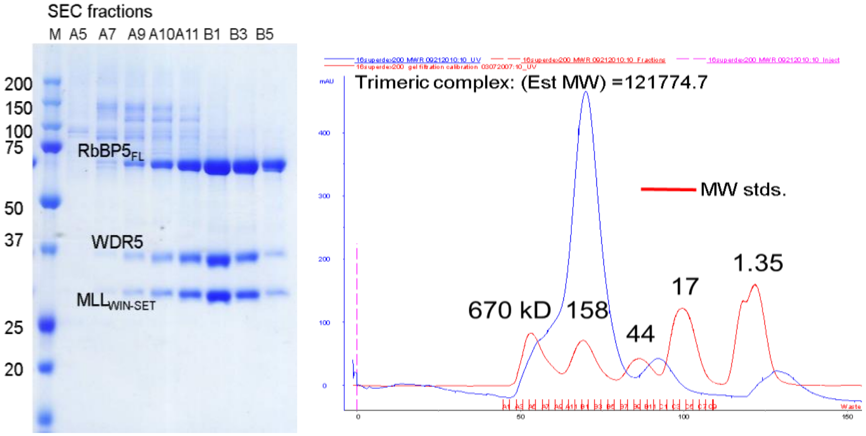


**Initial Purification using SEC**

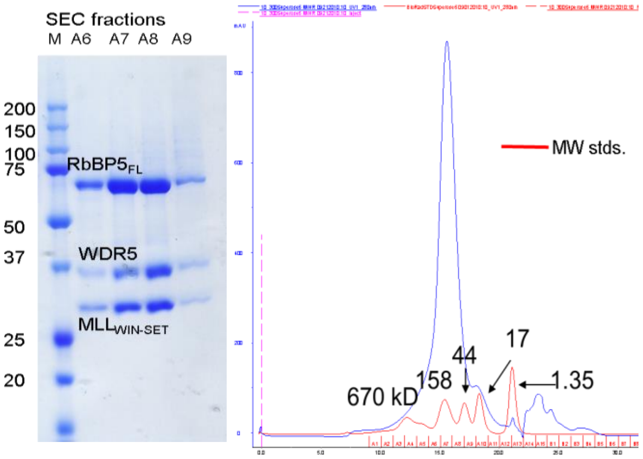


**Rerun SEC immediately after concentrating the trimer**

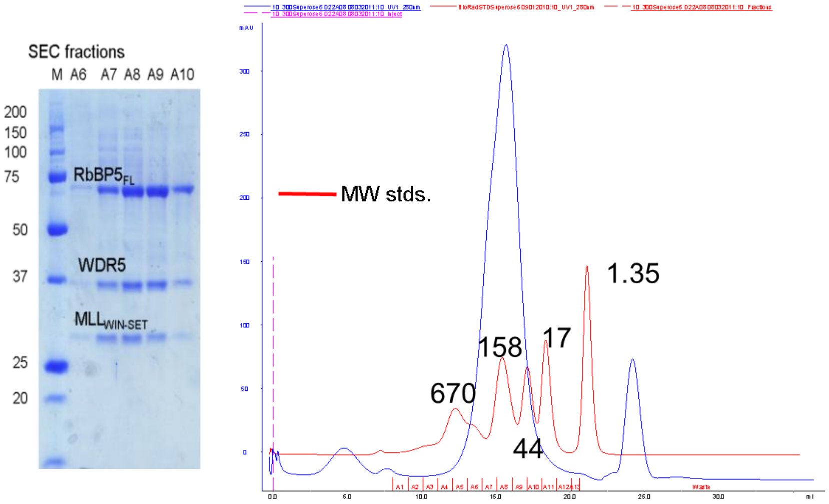


**Rerun SEC after a freeze-thaw cycle**

# Figure S6.

**Figure S6.**

**Structural characterization of WDR5-RbBP5-MLL1$_{WIN\text{-}SET}$. (A)** Rg-based (left) and Vc-based (right) Kratky plots of SAXS data for WDR5-RbBP5-MLL1$_{WIN\text{-}SET}$ (black), WDR5$_{WD40}$ (blue), MLL1$_{WIN\text{-}SET}$ (green), and RbBP5 (red). SAXS data indicate a high degree of flexibility of the complex. (**B**) Normalized pair distance distribution functions P(r) calculated from SAXS data with GNOM (4). (**C**) Surface representation of the four most populated models in the optimal ensemble overlaid with the average *ab-initio* SAXS-predicted molecular envelope (grey mesh). WDR5, MLL1 and RbBP5 are colored in blue, green and red, respectively. (**D**) Ensemble distribution of Cα-Cα distances of lysine residues participating in the six cross-links between the structured domains of the complex. (**E**) Superposition of RbBP5$_{NTD}$-WDR5 from the four most populated models derived from coarse-grained models used in MD simulations. (**F**) Superposition of RbBP5$_{NTD}$-MLL1$_{WIN\text{-}SET}$ of the three models comprising 80% of the optimal ensemble demonstrate that this sub-complex has a well defined structural organization derived from coarse-grained models used in the MD simulations. (**G**) Molecular model of RbBP5$_{NTD}$-MLL1$_{WIN\text{-}SET}$ interaction. The RbBP5$_{NTD}$ surface is colored in pink. MLL1$_{WIN\text{-}SET}$ is shown as a cartoon diagram in pale green. The helix of the SET-I subdomain is highlighted in cyan. The RBS residues are shown in red. The RbBP5 residues that are in contact with the MLL1 SET domain are shown in grey. (**H**) Surface representation of the MLL1 SET domain (residues 3815-3969). The helix of the SET-I subdomain is shown in cyan. The RbBP5$_{NTD}$/RBS binding surface overlaps somewhat with the corresponding RbBP5$_{AS+ABM}$-ASH2L$_{SPRY}$ binding surface of the MLL3 SET domain. Residues at the RbBP5$_{NTD}$/RBS binding interface are shown in pink, while those that are involved in binding to RbBP5$_{AS+ABM}$-ASH2L$_{SPRY}$ in the MLL3 complex (6) are shown in yellow. Residues that participate in both interactions are displayed in red. Two GLY residues, that serve as the hinge points of SET-I rotation, are shown in blue. (**I**) Control experiments were performed to ensure that the WDR5-RbBP5-MLL1$_{WIN\text{-}SET}$ trimer remained stable and monodisperse following purification. After initially purifying the trimer by size exclusion chromatography (SEC) (top left), we 1) repeated SEC immediately after concentrating the trimer-containing fractions (top right), and 2) repeated SEC after performing a freeze-thaw cycle with purified trimer (bottom left). In both cases, the species re-eluted at a consistent SEC elution volume indicating that it remained intact, and there was no indication of aggregation. We performed these controls to ensure samples remained in good condition following their preparation in Toronto and shipment frozen to Illinois and Switzerland for SAXS and XL-MS data collection, respectively.

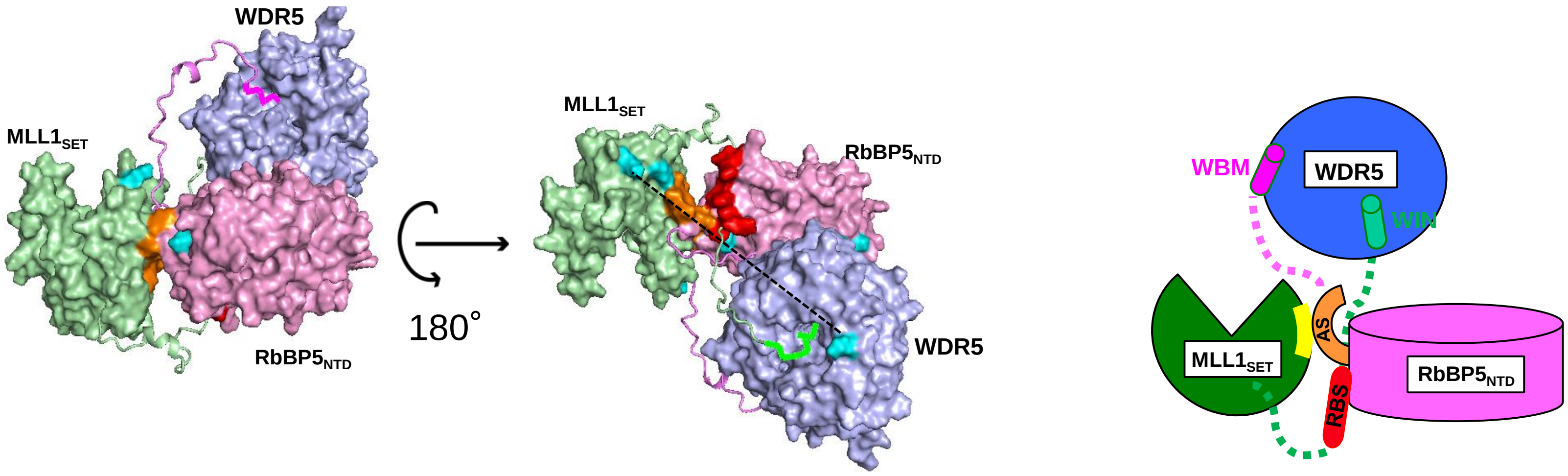


**Figure S7**. **Minor population of conformers in the optimal ensemble of the MLL1 trimer where there is dual NTD/AS RbBP5 interaction with MLL1**. The model is shown by a surface representation of the globular regions and a backbone trace for the flexible ones. For clarity, the CT of RbBP5 (i.e. $RbBP5_{382\text{-}538}$) is not shown and the residues from the AS are in dark orange, WIN in green, WBM in violet and RBS in red. The cross-link between $WDR5_{46}$ and $MLL1_{3846}$ that is inconsistent with the model is shown by a dashed black line (and the lysine residues are shown in cyan). Schematic representation of the domain arrangement and critical motif/segment interactions are shown to the right.

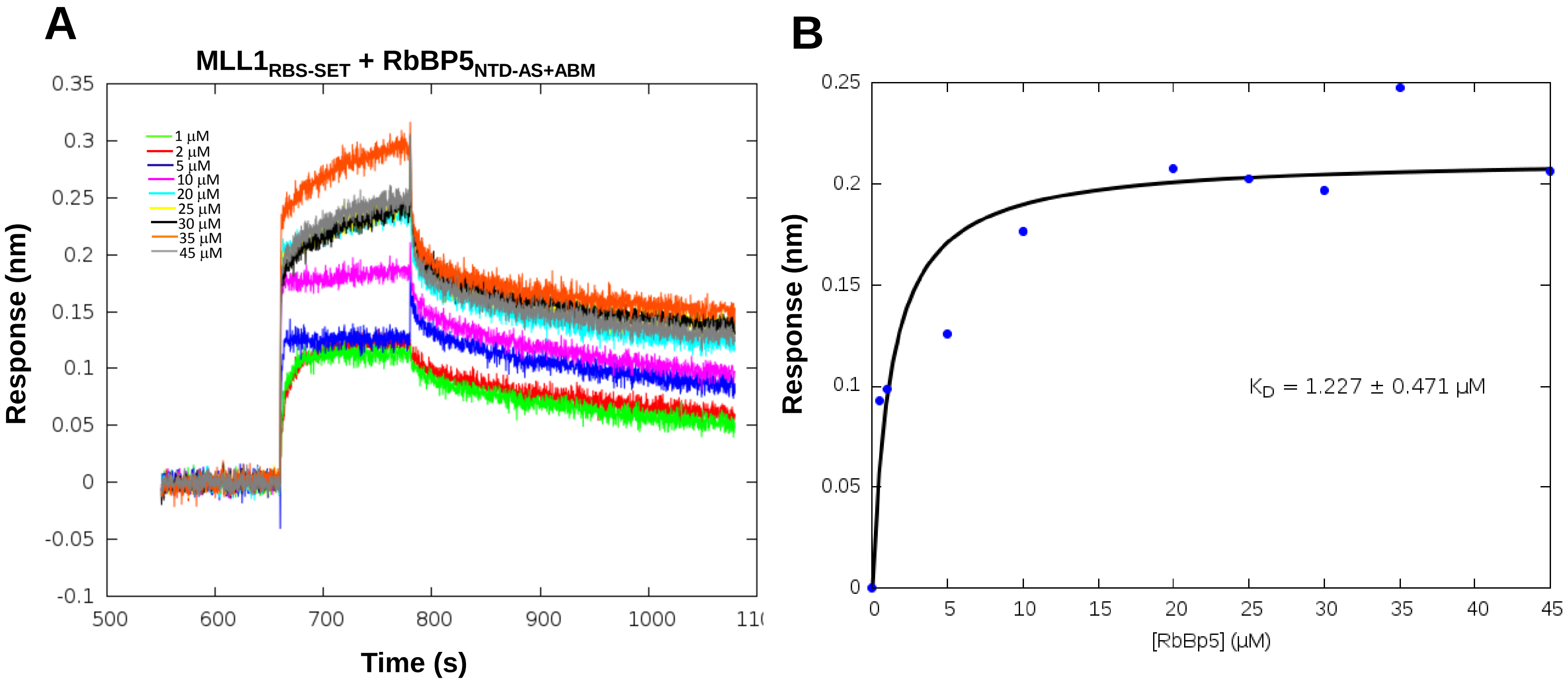


**Figure S8**. (**A**) BLI sensorgrams for the binding of $RbBP5_{NTD\text{-}AS+ABM}$ to sensor-immobilized GST-tagged $MLL1_{RBS\text{-}SET}$, (**B**) and the corresponding steady-state binding curve. The sensorgrams exhibit incomplete dissociation phases, and at higher ligand concentrations, non-steady-state binding behavior. This may be due to ligand aggregation in the presence of sensor-bound $MLL1_{RBS\text{-}SET}$.

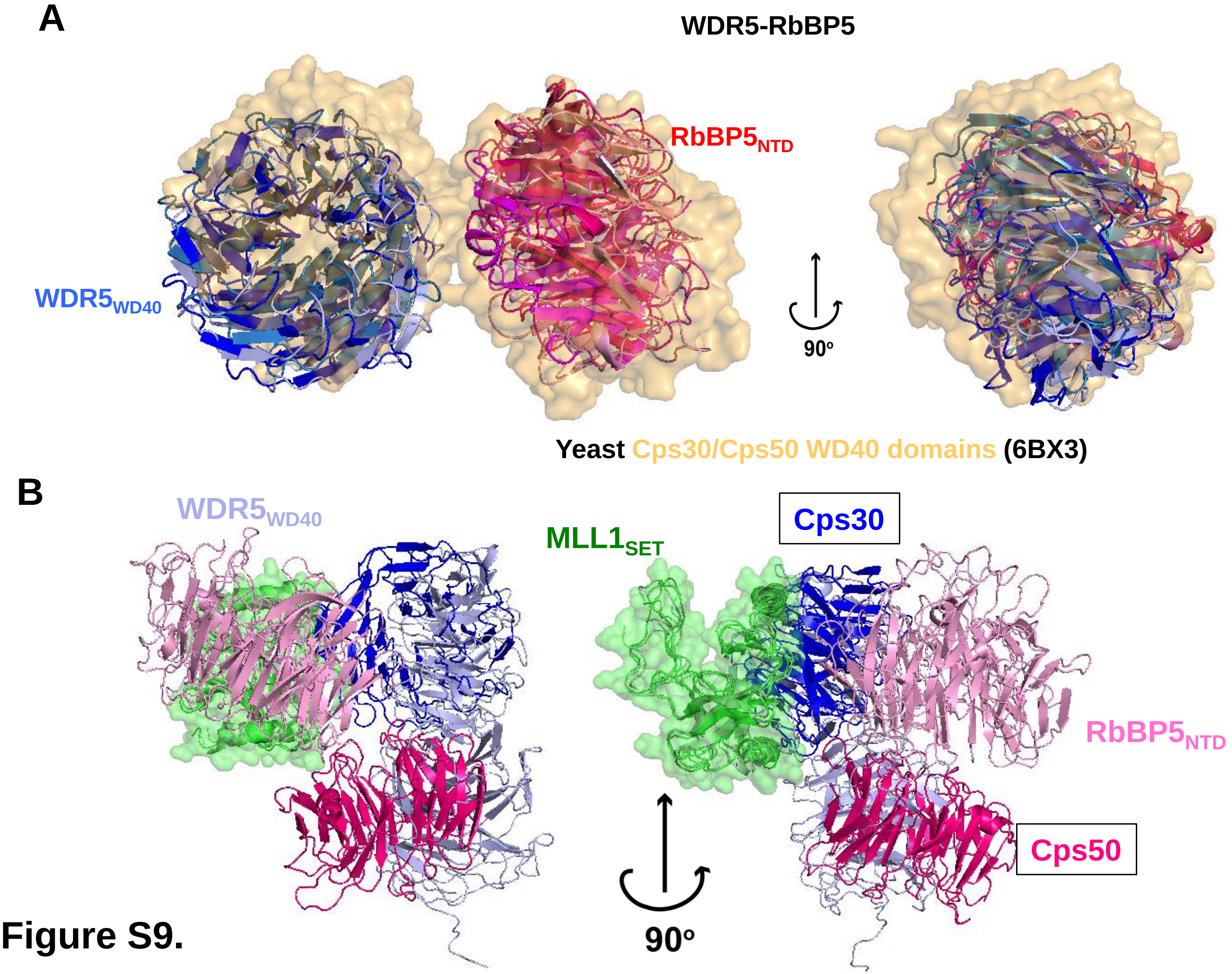
A
WDR5-RbBP5
RbBP5$_{NTD}$
WDR5$_{WD40}$
90°
Yeast Cps30/Cps50 WD40 domains (6BX3)
B
WDR5$_{WD40}$
MLL1$_{SET}$
Cps30
RbBP5$_{NTD}$
Cps50
90°

Figure S9.

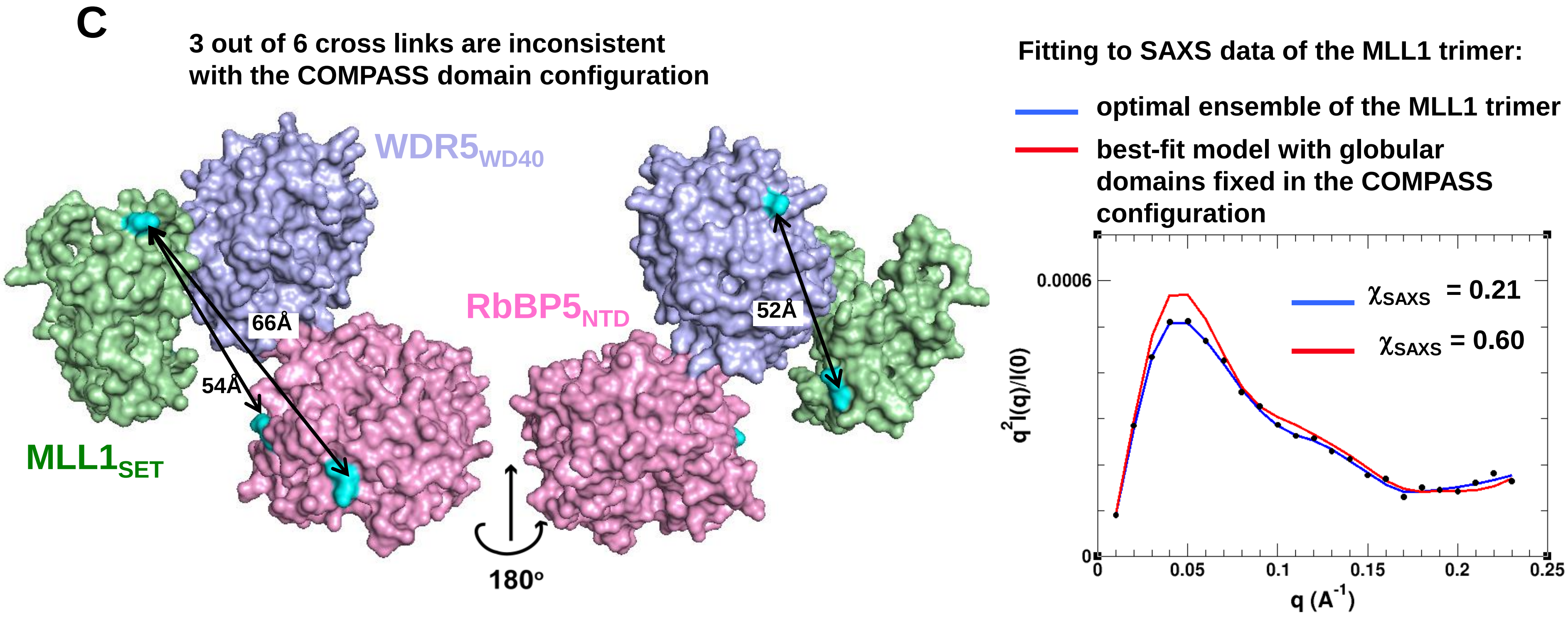


**Figure S9. Comparison of MLL1 trimer with the yeast COMPASS. (A)** Comparison of the relative configuration of $WDR5_{WD40}$ and $RbBP5_{NTD}$ domains in the SES optimal ensemble of WDR5-RbBP5 (see **Fig 3D**) with the configuration of WD40 domains of Cps30 and Cps50 in the cryo-EM structure of the yeast COMPASS (*PDBID*: 6BX3) (5). WDR5 (blue) and RbBP5 (pink) are in cartoon format while the crystal structure is shown by transparent surface representation (orange). (**B**) Comparison of the relative configuration of $MLL1_{SET}$, $WDR5_{WD40}$, and $RbBP5_{NTD}$ domains in our timer model (see **Fig 6**) with their position (of corresponding orthologues) in the cryo-EM structure of COMPASS (*PDBID*: 6BX3). The SET domains are superimposed. Yeast SET1 is shown in green by surface representation, while human $MLL1_{SET}$ is in green cartoon. $WDR5_{WD40}$ (pale blue), $RbBP5_{NTD}$ (pink), $Cps30_{WD40}$ (blue), and $Cps50_{WD40}$ (red) are shown by cartoon diagram. (**C**) The globular domain configuration in COMPASS is not consistent with SAXS and cross-link data of the MLL1 trimer. The three cross-links between globular domains that are inconsistent with the COMPASS configuration are shown by solid lines on the left. Kratky plot (right) of the experimental SAXS profile (black circles) and the ensemble averaged theoretical profiles for the MLL1 trimer (blue) and COMPASS configuration (red).

**REFERENCES**

1. Franke,D. and Svergun,D.I. (2009) DAMMIF, a program for rapid ab-initio shape determination in small-angle scattering. J. Appl. Crystallogr., 42, 342–346.
2. Rohl,C.A., Strauss,C.E.M., Misura,K.M.S. and Baker,D. (2004) Protein structure prediction using Rosetta. Methods Enzymol., 383, 66–93.
3. Cilia,E., Pancsa,R., Tompa,P., Lenaerts,T. and Vranken,W.F. (2013) From protein sequence to dynamics and disorder with DynaMine. Nat. Commun., 4, 2741.
4. Feigin,L.A. and Svergun,D.I. (1987) Structure analysis by small-angle X-ray and neutron scattering. In. Plenum Press.
5. Qu,Q., Takahashi,Y.-H., Yang,Y., Hu,H., Zhang,Y., Brunzelle,J.S., Couture,J.-F., Shilatifard,A. and Skiniotis,G. (2018) Structure and Conformational Dynamics of a COMPASS Histone H3K4 Methyltransferase Complex. Cell, 174, 1117–1126.e12.
6. Li,Y., Han,J., Zhang,Y., Cao,F., Liu,Z., Li,S., Wu,J., Hu,C., Wang,Y., Shuai,J., et al. (2016) Structural basis for activity regulation of MLL family methyltransferases. Nature, 530, 447–452.
